# ﻿Thirteen species of jumping spiders from northern Vietnam (Araneae, Salticidae)

**DOI:** 10.3897/zookeys.1148.98271

**Published:** 2023-02-16

**Authors:** Cheng Wang, Shuqiang Li, Dinh-Sac Pham

**Affiliations:** 1 College of Agriculture and Forestry Engineering and Planning, Guizhou Provincial Key Laboratory of Biodiversity Conservation and Utilization in the Fanjing Mountain Region, Tongren University, Tongren 554300, Guizhou, China Tongren University Tongren China; 2 Institute of Zoology, Chinese Academy of Sciences, Beijing 100101, China Institute of Zoology, Chinese Academy of Sciences Beijing China; 3 Vietnam National Museum of Nature (VNMN), Vietnam Academy of Science and Technology (VAST), 18 Hoang Quoc Viet, Cau Giay, Hanoi, Vietnam Vietnam National Museum of Nature Hanoi Vietnam

**Keywords:** Morphology, new taxa, salticid, Southeast Asia, taxonomy

## Abstract

A new genus and thirteen species of jumping spiders from northern Vietnam are reported. *Zabka***gen. nov.** is erected to accommodate two species transferred from *Euophrys* Blackwall,1841, including the generotype, *Z.cooki* (Żabka, 1985), **comb. nov.**, and *Z.xuyei* (Lin & Li, 2020), **comb. nov.** Twelve new species are described: *Chinattuscrewsae***sp. nov.** (♂♀), *C.logunovi***sp. nov.** (♂♀), *Eupoamaidinhyeni***sp. nov.** (♂♀), *E.maddisoni***sp. nov.** (♂♀), *E.ninhbinh***sp. nov.** (♂), *E.zabkai***sp. nov.** (♂♀), *Indopadillacuc***sp. nov.** (♂♀), *Synagelidesani***sp. nov.** (♂♀), *S.mii***sp. nov.** (♂♀), *S.pengi***sp. nov.** (♀), *S.sancha***sp. nov.** (♂♀), and *Yaginumaellahagiang***sp. nov.** (♂). The unknown male of *Zabkacooki* is described for the first time. Diagnostic photos of the habitus and copulatory organs are provided.

## ﻿Introduction

The interactions of complex topographic, climatic, and ecological factors through time are responsible for the high levels of species diversity and endemism in Vietnam, one of eleven Southeast Asian countries (Liu et al. 2010). Taxonomic study of jumping spiders from Vietnam began at the end of the 19^th^ century, but little was known until the systematic work of Żabka (1985) who described 100 species, including 51 species new to science. Later, a series of publications by Dr. Dmitri V. Logunov greatly increased the knowledge of the diversity of Vietnamese salticid fauna (Logunov 2001, 2008, 2021; Logunov and Marusik 2014; Logunov and Jäger 2015). However, a high number of endemic species remains known only from a single sex (Metzner 2023; WSC 2023). To date, at least 135 species in 67 genera have been recorded, but the true diversity of jumping spiders from this area remains insufficiently known and is likely to be much greater (Logunov 2021; Hoang et al. 2022).

This study presents data collected from three National Parks and Ha Giang National Forest in northern Vietnam (Liu et al. 2010; Lin et al. 2023) and is goal to erect a new genus, to describe 12 new species of five genera and the unknown male of *Zabkacooki* (Żabka, 1985), comb. nov.

## ﻿Materials and methods

Specimens were collected by sieving, pitfall trap, or hand collecting and were preserved in 75% ethanol for morphological study. All specimens are deposited in the Institute of Zoology, Chinese Academy of Sciences (**IZCAS**) in Beijing, China. Methods follow those of Wang and Li (2021).

All measurements are given in millimeters. Leg measurements are given as: total length (femur, patella, tibia, metatarsus, tarsus). References to figures in the cited papers are listed in lowercase type (fig. or figs), and figures in this paper are noted with an initial capital (Fig. or Figs).

Abbreviations used in the text and figures are as follows: **AERW** anterior eye row width; **AME** anterior median eye; **ALE** anterior lateral eye; **AG** accessory gland; **AR** atrial ridge; **At** atrium; **CD** copulatory duct; **CO** copulatory opening; **CTA** compound terminal apophysis; **DCA** dorsal cymbial apophysis; **DP** dorsal epigynal plate; **dTA** division of terminal apophysis; **DTA** dorsal tibial apophysis; **dRTA** dorso-retrolateral tibial apophysis; **E** embolus; **ED** embolic division; **EFL** eye field length; **FD** fertilization duct; **H** epigynal hood; **MA** median apophysis; **MS** median septum; **PCA** prolateral cymbial apophysis; **PERW** posterior eye row width; **PL** posterior lobe; **PME** posterior median eye; **PLE** posterior lateral eye; **PTA** prolateral tibial apophysis; **RCA** retrolateral cymbial apophysis; **RFA** retrolateral femoral apophysis; **RPA** retrolateral patellar apophysis; **RTA** retrolateral tibial apophysis; **S** spermatheca; **SD** sperm duct; **TA** terminal apophysis; **VPA** ventral patellar apophysis; **VTA** ventral tibial apophysis.

## ﻿Taxonomy

### ﻿Genus Chinattus

Taxon classificationAnimaliaAraneaeSalticidae

Logunov, 1999

ABA3B963-392B-51D2-AFE5-83F325BDA5C5

#### Type species.

*Habrocestoidesszechwanensis* Prószyński, 1992 from China.

#### Comments.

*Chinattus* is placed in the tribe Hasariini together with thirteen other genera, and is represented by 20 species mainly distributed in Asia, as well as presents the highest diversity in China (Maddison 2015; WSC 2023). The genus is rather well studied due to all of its species having diagnostic drawings, but more than one-third of these species are known only from a single sex (WSC 2023). To date, only two species have been recorded from Vietnam (WSC 2023). In the present work, we place the two new species described below into *Chinattus* due to the similarity of habitus and copulatory organs. However, it is worth mentioning that they are also different from other *Chinattus* species by the absence of a basal epigynal plate and its round internal structure, and having retromarginal cheliceral fissident tooth with at least five cusps (vs. the presence of basal epigynal plate with a round internal structure and having a single retromarginal cheliceral tooth in the generotype and its congeners; see Metzner 2023), and so, the generic position of those two species may need further confirmation. Moreover, *Chinattus* presents very similar habitus and copulatory organs with *Jajpurattus* Prószyński, 1992, and the relationship between these two genera may also need further attention.

### ﻿ Chinattus crewsae sp. nov.

Taxon classificationAnimaliaAraneaeSalticidae

2E13484B-A9C2-5E81-A508-9760D47FABD5

https://zoobank.org/EE86D8F4-B52C-47F6-9567-EFD5476C9727

Figs 1 , 2

#### Type material.

***Holotype*** ♂ (IZCAS-Ar44157), Vietnam: Ha Giang Province: Ha Giang National Forest, 14.V.2002, D.S. Pham leg. ***Paratypes*** 12♂4♀ (IZCAS-Ar44158–44173), same data as holotype; 7♂5♀ (IZCAS-Ar44174–44185), same locality and collector, 14.VIII.2002.

#### Etymology.

This specific name is a patronym in honor of Sarah Crews (San Francisco, USA), a leading specialist on the taxonomy of Selenopidae worldwide; noun (name) in genitive case.

#### Diagnosis.

**Figure 1.**
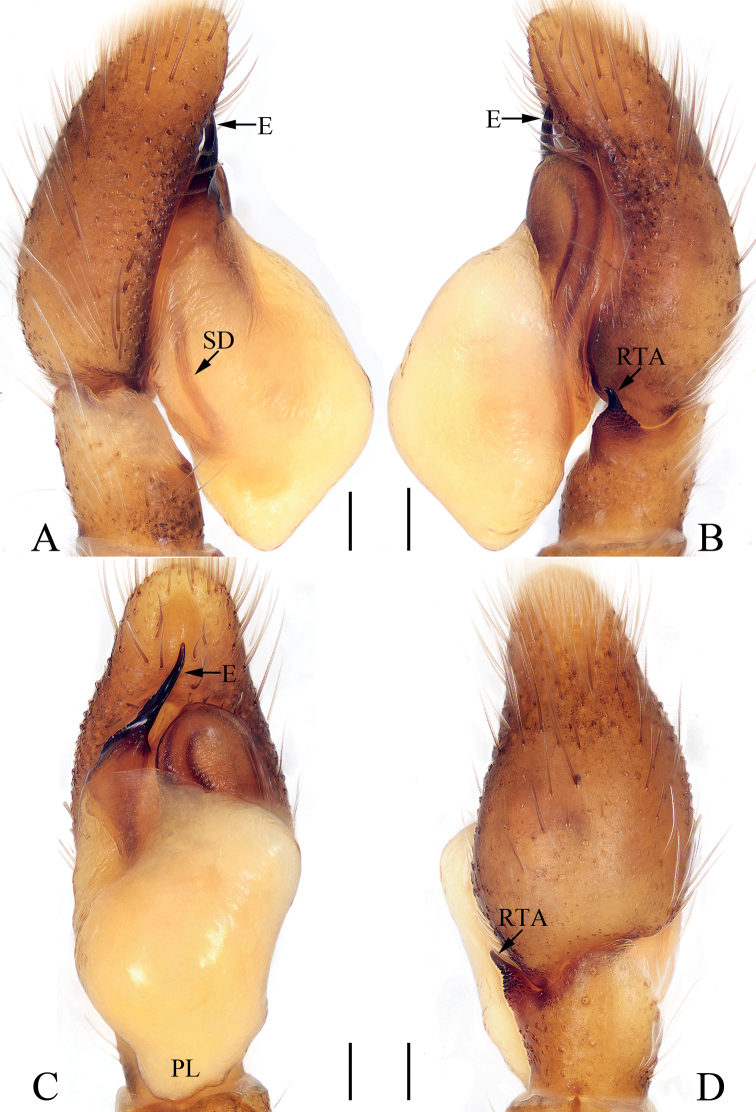
Male palp of *Chinattuscrewsae* sp. nov., holotype **A** prolateral **B** retrolateral **C** ventral **D** dorsal. Scale bars: 0.1 mm. Abbreviations: E – embolus; PL – posterior lobe; RTA – retrolateral tibial apophysis; SD – sperm duct.

**Figure 2.**
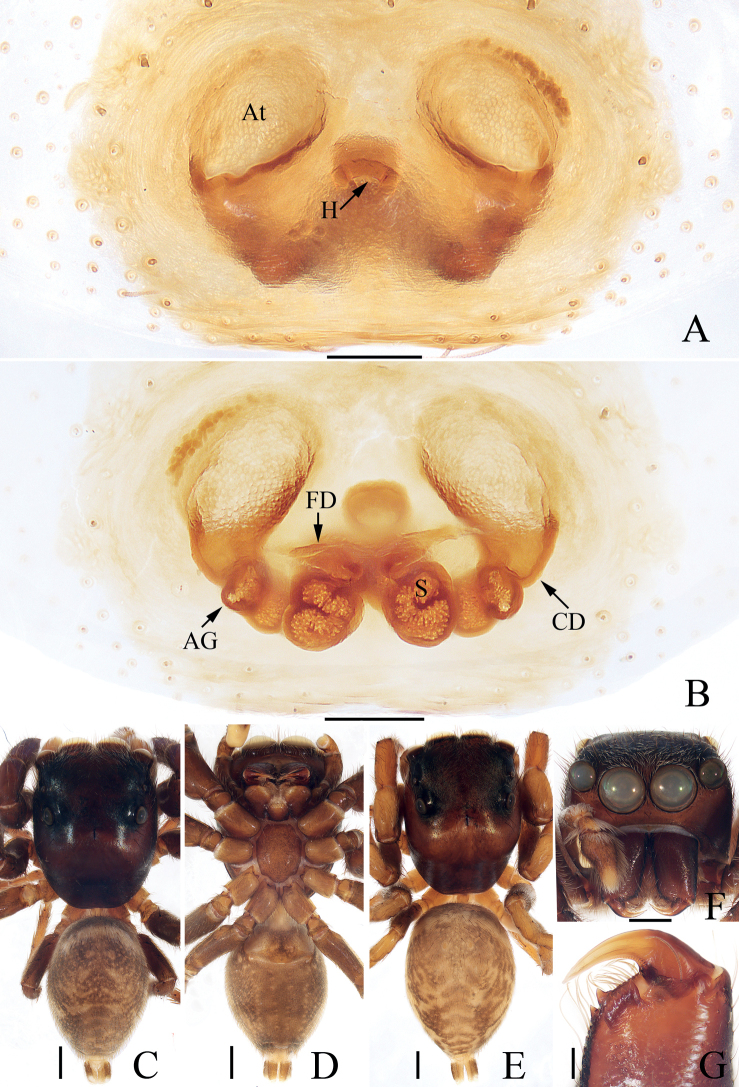
*Chinattuscrewsae* sp. nov., male holotype and female paratype **A** epigyne, ventral **B** vulva, dorsal **C** holotype habitus, dorsal **D** ditto, ventral **E** female paratype habitus, dorsal **F** holotype carapace, frontal **G** holotype chelicera, posterior. Scale bars: 0.1 mm (**A, B, G**); 0.5 mm (**C–F**). Abbreviations: AG – accessory gland; At – atrium; CD – copulatory duct; FD – fertilization duct; H – epigynal hood; S – spermatheca.

*Chinattuscrewsae* sp. nov. resembles *C.furcatus* (Xie, Peng & Kim, 1993) in the general shape of copulatory organs, but it can be easily distinguished by the following: (1) the RTA is acutely narrowed medially, and curved inward at distal end in retrolateral view (Fig. 1B), whereas it is tapered, not curved in *C.furcatus* (Peng and Xie 1995: fig. 3); (2) the copulatory openings open anteriorly (Fig. 2A), whereas they open bilaterally in *C.furcatus* (Peng and Xie 1995: fig. 5); (3) the epigynal hood is square, located medially (Fig. 2A), whereas almost triangular, located posteriorly in *C.furcatus* (Peng and Xie 1995: fig. 5). The female of this new species also resembles *Hasariusorientalis* (Żabka, 1985) in having similar epigyne and multifurcated retromarginal cheliceral fissidental tooth, but it can be easily distinguished by the following: (1) the copulatory openings are located anteriorly (Fig. 2A), whereas they are located posteriorly in *H.orientalis* (Żabka 1985: fig. 214); (2) the epigynal hood is located medially (Fig. 2A), whereas it is located postero-marginally in *H.orientalis* (Żabka 1985: fig. 214).

#### Description.

**Male** (Figs 1, 2C, D, F, G). Total length 4.76. Carapace 2.41 long 1.93 wide. Abdomen 2.17 long, 1.59 wide. Clypeus 0.12 high. Eye sizes and inter-distances: AME 0.58, ALE 0.36, PLE 0.30, AERW 1.86, PERW 1.70, EFL 1.16. Legs: I 5.14 (1.50, 1.01, 1.25, 0.88, 0.50), II 4.37 (1.33, 0.78, 0.88, 0.93, 0.45), III 5.37 (1.88, 0.83, 1.13, 1.05, 0.48), IV 4.75 (1.50, 0.63, 1.01, 1.13, 0.48). Carapace almost square, red-brown to dark brown, setose; fovea dark, longitudinal, bar-shaped. Chelicerae red-brown to dark-brown, with two promarginal teeth and one retromarginal fissidental tooth with five to eight cusps. Endites red-brown, paler the inner margins, broadened distally. Labium colored as endites. Sternum longer than wide, almost shield-shaped. Legs yellow to dark brown. Abdomen oval, dorsum brown, with arc-shaped white stripe of setae at anterior sub-margin, followed by a pair of muscle depressions, and three triangular brown patches encircled by discontinuous, yellow patches; venter brown, with longitudinal dotted lines. Palp (Fig. 1A–D): tibia slightly longer than wide; RTA acutely narrowed, and strongly sclerotized at distal half, slightly curved inward at distal end, with broadened medio-proximal portion bearing dense processes; cymbium setose; bulb elongated, swollen, with blunt posterior lobe extending posteriorly; embolus originates from antero-prolateral portion of bulb, twisted, extending anteriorly, with rather pointed tip.

**Female** (Fig. 2A, B, E). Total length 5.78. Carapace 2.75 long 2.13 wide. Abdomen 2.81 long, 2.03 wide. Clypeus 0.14 high. Eye sizes and inter-distances: AME 0.59, ALE 0.37, PLE 0.31, AERW 2.01, PERW 1.81, EFL 1.25. Legs: I 4.65 (1.42, 0.93, 1.10, 0.70, 0.50), II 4.36 (1.38, 0.83, 0.95, 0.70, 0.50), III 5.88 (2.01, 0.83, 1.43, 1.13, 0.48), IV 5.18 (1.60, 0.68, 1.15, 1.25, 0.50). Habitus (Fig. 2E) similar to that of male except paler, with one retromarginal cheliceral fissidental tooth with six cusps. Epigyne (Fig. 2A, B): wider than long, with sub-square central hood, and pair of anterolateral oval atria; copulatory openings beneath the posterior-most of atrial margins; copulatory ducts twisted, with proximal, short accessory glands; spermathecae indistinct; fertilization ducts lamellar, extending transversely.

#### Distribution.

Known only from the type locality in Ha Giang Province, Vietnam.

### ﻿ Chinattus logunovi sp. nov.

Taxon classificationAnimaliaAraneaeSalticidae

DC590567-F626-59EE-880D-1BFEE8E88D05

https://zoobank.org/59B5E74A-02BE-4F2F-98FA-B9961B414A63

Figs 3 , 4

#### Type material.

***Holotype*** ♂ (IZCAS-Ar44186), Vietnam: Vinh Phuc Province: Tam Dao National Park, Acacia Plantation, 1–30.III.2008, D.S. Pham leg. ***Paratypes*** 2♂1♀ (IZCAS-Ar44187–44189), same data as holotype; 1♂1♀ (IZCAS-Ar44190–44191), Tam Dao National Park, 1–30.I.2008, same collector.

#### Etymology.

The specific name is a patronym in honor of Dmitri V. Logunov (Manchester, UK), a leading arachnologist in jumping spiders, who has contributed significantly to the taxonomy of jumping spiders from Vietnam; noun (name) in genitive case.

#### Diagnosis.

**Figure 3.**
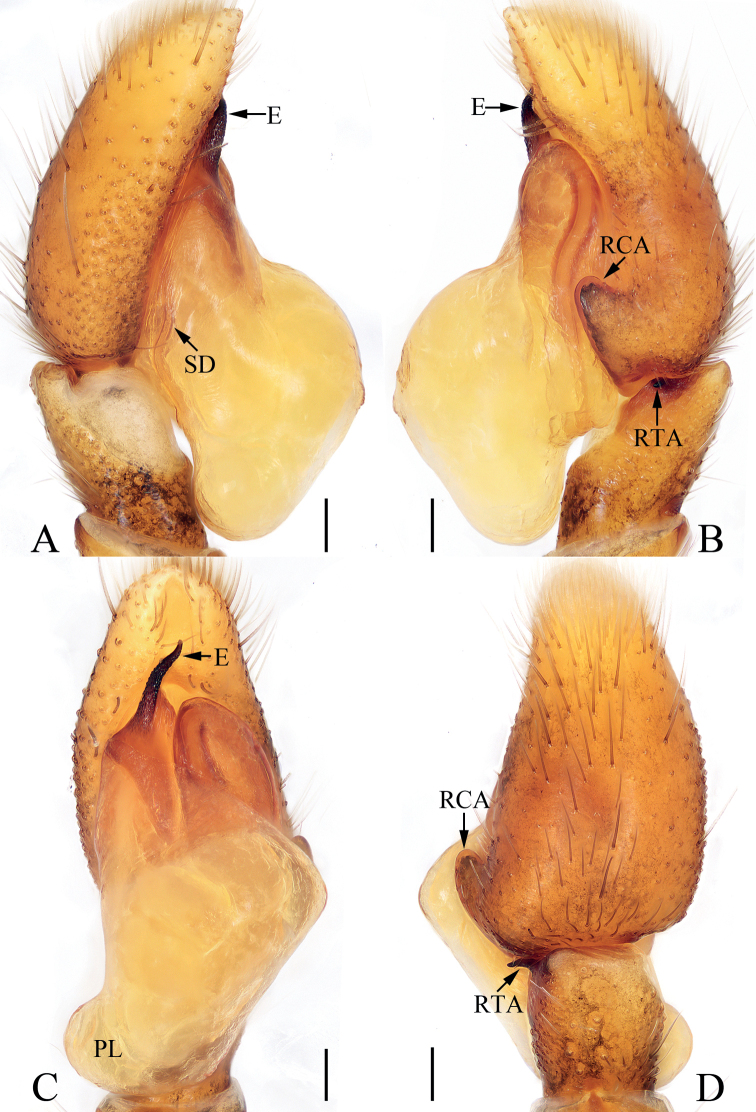
Male palp of *Chinattuslogunovi* sp. nov., holotype **A** prolateral **B** retrolateral **C** ventral **D** dorsal. Scale bars: 0.1 mm. Abbreviations: E – embolus; PL – posterior lobe; RCA – retrolateral cymbial apophysis; RTA – retrolateral tibial apophysis; SD – sperm duct.

**Figure 4.**
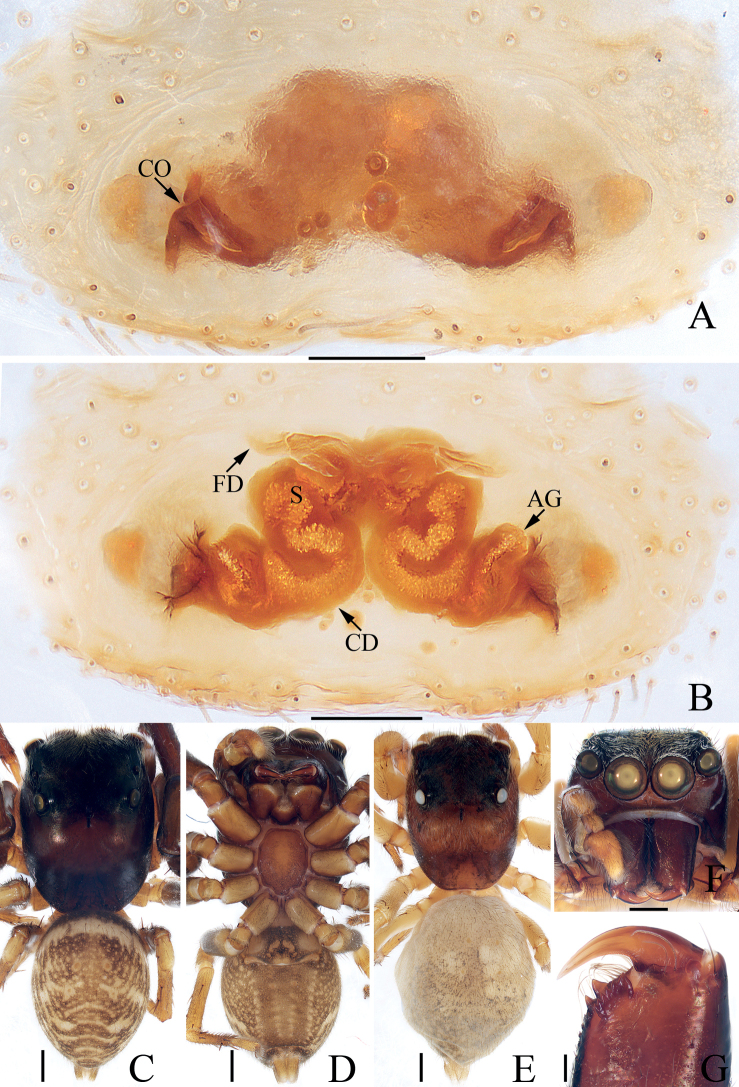
*Chinattuslogunovi* sp. nov., male holotype and female paratype **A** epigyne, ventral **B** vulva, dorsal **C** holotype habitus, dorsal **D** ditto, ventral **E** female paratype habitus, dorsal **F** holotype carapace, frontal **G** holotype chelicera, posterior. Scale bars: 0.1 mm (**A, B, G**); 0.5 mm (**C–F**). Abbreviations: AG – accessory gland; CD – copulatory duct; CO – copulatory opening; FD – fertilization duct; S – spermatheca.

The male of *Chinattuslogunovi* sp. nov. can be easily distinguished from other congeners by the presence of RCA (Fig. 3B). The female of this new species resembles that of *C.prabodhi* Basumatary, Das, Caleb & Brahma, 2020 by the absence of epigynal plate, but it can be easily distinguished by the spermathecae, which are anterior to copulatory openings (Fig. 4B), whereas almost as level as copulatory openings in *C.prabodhi* (Basumatary et al. 2020: figs 8–11), and by the accessory glands of copulatory ducts, which are anteriorly extending (Fig. 4B), whereas posteriorly extending in *C.prabodhi* (Basumatary et al. 2020: fig. 11).

#### Description.

**Male** (Figs 3, 4C, D, F, G). Total length 5.45. Carapace 2.95 long 2.23 wide. Abdomen 2.59 long, 1.91 wide. Clypeus 0.14 high. Eye sizes and inter-distances: AME 0.59, ALE 0.36, PLE 0.30, AERW 1.94, PERW 1.73, EFL 1.14. Legs: I 7.52 (2.13, 1.33, 1.88, 1.43, 0.75), II 4.81 (1.45, 0.88, 1.10, 0.88, 0.50), III 6.21 (2.08, 0.93, 1.45, 1.25, 0.50), IV 5.74 (1.68, 0.93, 1.20, 1.38, 0.55). Carapace sub-square, slightly narrowed anteriorly, red-brown to dark brown, covered with setae, denser anteriorly, with fan-shaped, red-brown area medially; fovea bar-shaped, longitudinal, dark. Chelicerae red-brown, with two promarginal teeth and one retromarginal fissidental tooth with six cusps. Endites broadened distally, bearing dense setae at distal half on inner margins. Labium red-brown, almost linguiform, paler distally. Sternum colored as labium, longer than wide, with straight anterior margin. Legs yellow to brown, spinous. Abdomen almost oval, dorsum brown, spotted, with arc-shaped, pale stripe anteriorly, two pairs of muscle depressions, several pairs of irregularly shaped spots mediolaterally, several wave-shaped or straight, transverse stripes posteromedially; venter colored as dorsum, with two pairs of pale, longitudinal dotted lines. Palp (Fig. 3A–D): tibia longer than wide, with short, strongly sclerotized retrolateral apophysis curved medially, and with rather pointed tip; cymbium longer than wide, with flat, apically blunt baso-retrolateral apophysis; bulb longer than wide, swollen medio-posteriorly, with semi-circular posterior lobe extending prolaterally; embolus strongly sclerotized, slightly curved, originates from antero-prolateral portion of bulb, with rather pointed tip, directed anteriorly in ventral view.

**Female** (Fig. 4A, B, E). Total length 5.56. Carapace 2.52 long 1.96 wide. Abdomen 3.01 long, 2.44 wide. Clypeus 0.15 high. Eye sizes and inter-distances: AME 0.54, ALE 0.34, PLE 0.29, AERW 1.66, PERW 1.57, EFL 1.03. Legs: I 4.76 (1.45, 0.88, 1.13, 0.80, 0.50), II 4.09 (1.13, 0.80, 1.01, 0.70, 0.45), III 5.64 (1.95, 0.88, 1.18, 1.13, 0.50), IV 5.29 (1.68, 0.70, 1.13, 1.25, 0.53). Carapace similar to that of male except paler. Chelicerae with one retromarginal fissidental tooth with seven cusps. Abdomen pale brown, with two pairs of muscle depressions medially. Epigyne (Fig. 4A, B): wider than long, with oval structure (maybe a transformation of epigynal hood) between copulatory openings; copulatory openings posterolaterally located, opened laterally; copulatory ducts thick, twisted, strongly curved, with proximal accessory glands; spermathecae indistinct; fertilization ducts lamellar, transversely extending.

#### Distribution.

Known only from the type locality in Vinh Phuc Province, Vietnam.

### ﻿Genus Eupoa

Taxon classificationAnimaliaAraneaeSalticidae

Żabka, 1985

8DAC5282-624E-5878-A0A3-DD7AFDB398D3

#### Type species.

*Eupoaprima* Żabka, 1985 from Vietnam.

#### Comments.

*Eupoa* is placed into the subfamily Eupoinae with other three genera (Maddison 2015; Metzner 2023) and contains 14 leaf-litter dwellers mainly distributed in Southeast Asia (WSC 2023). The genus has high endemism and includes 11 species known only from a single country (WSC 2023). To date, three species have been recorded from Vietnam, of which two are endemic and one of those is known only from females.

### ﻿ Eupoa maidinhyeni sp. nov.

Taxon classificationAnimaliaAraneaeSalticidae

24CC7EE1-0B92-5B83-83E2-8B3B0A0634A4

https://zoobank.org/26326AF6-A19C-45DD-A65D-F61D976C5309

Figs 5 , 6

#### Type material.

***Holotype*** ♂ (IZCAS-Ar44192), Vietnam: Ninh Binh Province: Cuc Phuong National Park, Disturbed Forest (20°16.07'N, 105°42.04'E, ca. 250 m), 18.VIII.2007, D.S. Pham leg. ***Paratypes*** 1♀ (IZCAS-Ar44193), same data as holotype; 2♀ (IZCAS-Ar44194–44195), Cuc Phuong National Park (20°16.16'N, 105°41.32'E, ca. 250 m), 9.V.2007, same collector; 1♀ (IZCAS-Ar44196), Cuc Phuong National Park (20°23.12'N, 105°33.34'E, ca. 200 m), 7.VI.2007, same collector; 1♀ (IZCAS-Ar44197), Disturbed Forest (20°22.27'N, 105°33.05'E, ca. 340 m), 6.XI.2007, same collector; 1♂ (IZCAS-Ar44198), Cuc Phuong National Park (20°22.27'N, 105°33.05'E, ca. 200 m), 6.II.2008, same collector.

#### Etymology.

The specific name is after ichthyologist Mai Dinh Yen, born in 1933 in Ba Vi, Hanoi; noun (name) in genitive case.

#### Diagnosis.

**Figure 5.**
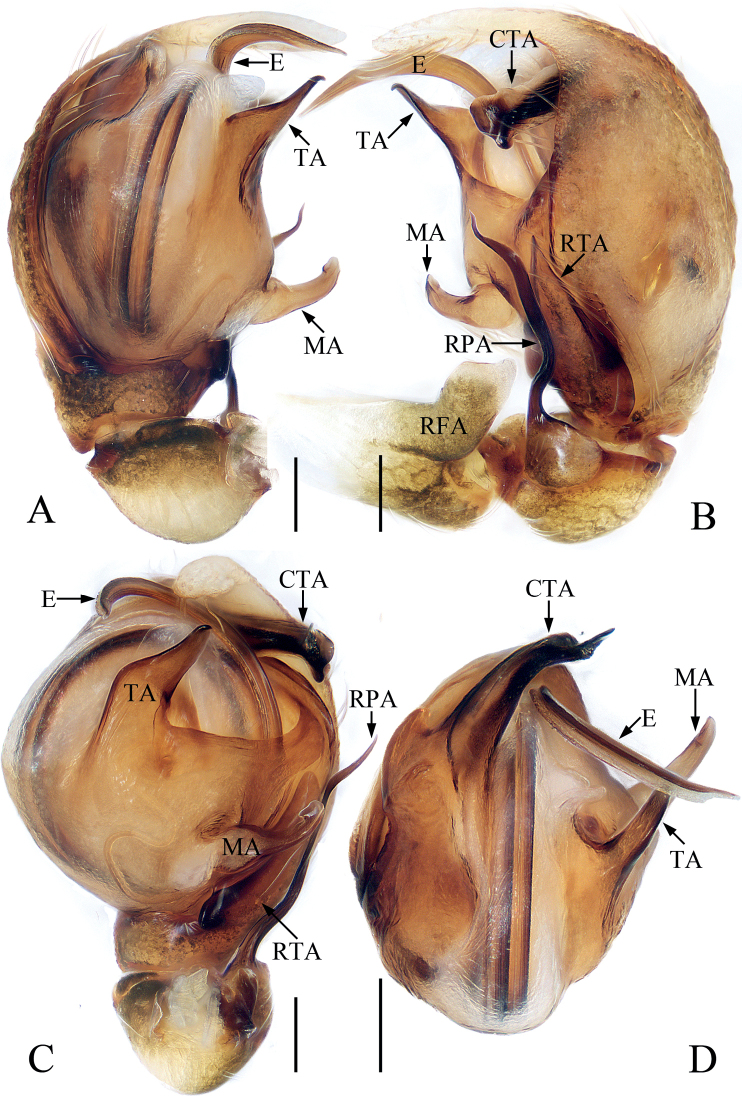
Male palp of *Eupoamaidinhyeni* sp. nov., holotype and paratype **A** holotype, prolateral **B** ditto, retrolateral **C** ditto, ventral **D** paratype bulb, apical. Scale bars: 0.1 mm. Abbreviations: E – embolus; CTA – compound terminal apophysis; MA – median apophysis; RFA – retrolateral femoral apophysis; RPA – retrolateral patellar apophysis; RTA – retrolateral tibial apophysis; TA – terminal apophysis.

**Figure 6.**
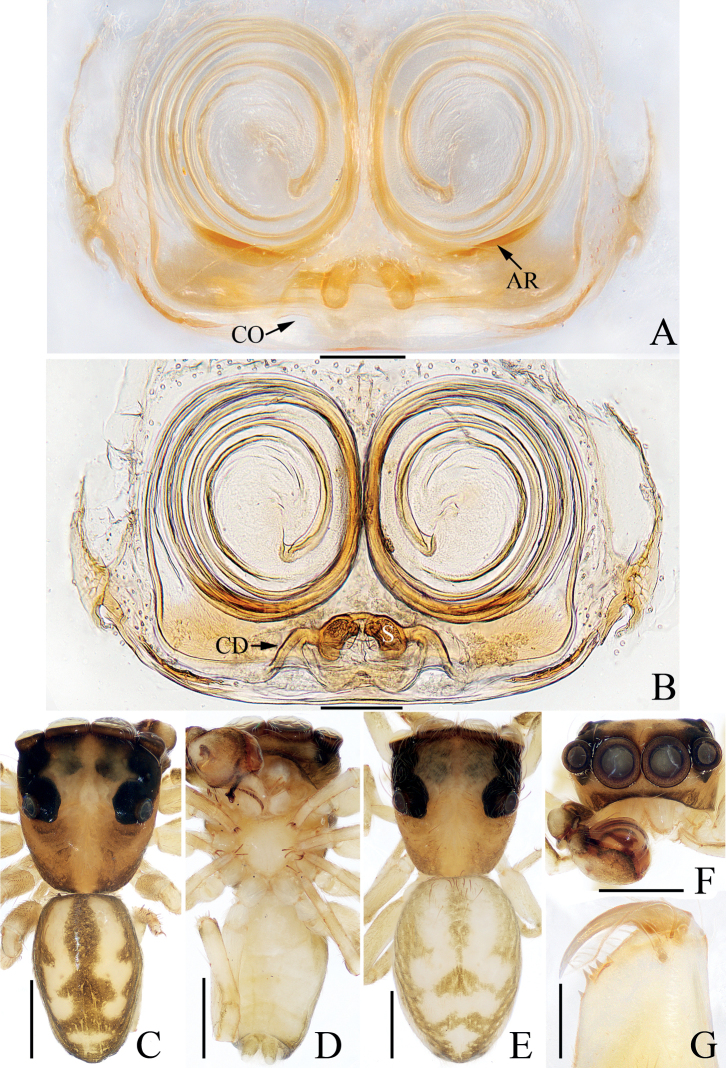
*Eupoamaidinhyeni* sp. nov., male holotype and female paratype **A** epigyne, ventral **B** vulva, dorsal **C** holotype habitus, dorsal **D** ditto, ventral **E** female paratype habitus, dorsal **F** holotype carapace, frontal **G** holotype chelicera, posterior. Scale bars: 0.1 mm (**A, B, G**); 0.5 mm (**C–F**). Abbreviations: AR – atrial ridge; CD – copulatory duct; CO – copulatory opening; S – spermatheca.

*Eupoamaidinhyeni* sp. nov. can be easily distinguished from other congeners by the well-developed CTA, which extends beyond the cymbial retromargin in ventral view, the thick, sickle-shaped embolus, and the circled atrial ridges (Figs 5C, 6A), whereas the CTA does not extend beyond the cymbial margin, the embolus is slender, flagelliform, and lacks similar circled atrial ridges in the other species (Metzner 2023).

#### Description.

**Male** (Figs 5, 6C, D, F, G). Total length 2.13. Carapace 1.05 long, 0.93 wide. Abdomen 1.09 long, 0.72 wide. Clypeus 0.05 high. Eye sizes and inter-distances: AME 0.30, ALE 0.21, PLE 0.18, AERW 0.99, PERW 0.85, EFL 0.57. Legs: I 1.81 (0.55, 0.28, 0.40, 0.38, 0.20), II 1.58 (0.50, 0.25, 0.33, 0.30, 0.20), III 1.61 (0.48, 0.25, 0.30, 0.38, 0.20), IV 2.14 (0.70, 0.28, 0.53, 0.43, 0.20). Carapace longer than wide, pale to dark yellow except the sides of eye field and posterior eyes bases dark, with pair of irregular dark patches medially on eye field, and longitudinal, central, yellow patch extending across thorax; fovea indistinct. Chelicerae pale, with two promarginal and four retromarginal teeth. Endites, sternum colored as chelicerae. Labium slightly darker than endites. Legs pale to yellow, with one ventral spine on tibiae I, and three pairs of ventral spines on metatarsi I. Abdomen elongated, dorsum pale yellow to green-brown, covered wholly by scutum, with pair of longitudinal, irregular, pale yellow stripes on ~ 3/4 of length, and separated by longitudinal, irregular, green brown central stripes and followed by transverse, pale yellow, posterior stripe; venter pale, without markings. Palp (Fig. 5A–D): femur longer than wide, with L-shaped disto-retrolateral apophysis; patella slightly longer than wide, with slender, curved, apically pointed retrolateral apophysis broadened proximally and more than half cymbial length in retrolateral view; tibia very short, with flat, broad retrolateral apophysis pointed apically; cymbium acutely tapered, strongly curved ventrally at distal 1/4; bulb almost round in ventral view; MA extending retrolaterally, with membranous base, forming hook at distal end; TA flat, slightly curved medially in ventral view, tapered to rather pointed tip distally; CTA strongly sclerotized, originates from antero-prolateral portion of bulb, extending retrolaterally and beyond retrolateral cymbial margin distally; embolus long, partly invisible, circled and sickle-shaped.

**Female** (Fig. 6A, B, E). Total length 2.44. Carapace 1.06 long, 0.96 wide. Abdomen 1.38 long, 0.96 wide. Clypeus 0.05 high. Eye sizes and inter-distances: AME 0.31, ALE 0.21, PLE 0.18, AERW 1.00, PERW 0.95, EFL 0.58. Legs: I 1.86 (0.55, 0.28, 0.45, 0.38, 0.20), II 1.63 (0.50, 0.25, 0.35, 0.33, 0.20), III 1.66 (0.50, 0.25, 0.33, 0.38, 0.20), IV 2.34 (0.75, 0.33, 0.58, 0.48, 0.20). Habitus (Fig. 6E) similar to that of male except paler, without dorsal abdominal scutum, and with three pairs of ventral spines on tibiae I. Epigyne (Fig. 6A, B): wider than long, with pair of round atria with circled, and arc-shaped ridges; copulatory openings posteriorly located, separated from each other by ~ 2× their width; copulatory ducts short, bent medially, connected to mediolateral margins of spermathecae; spermathecae elongate-oval.

#### Distribution.

Known only from the type locality in Ninh Binh Province, Vietnam.

### ﻿ Eupoa maddisoni sp. nov.

Taxon classificationAnimaliaAraneaeSalticidae

C35C2BCC-AB62-5C06-9427-15AF56A6CE22

https://zoobank.org/B701DBE1-71C0-4566-8505-28E1174B20BA

Figs 7 , 8

#### Type material.

***Holotype*** ♂ (IZCAS-Ar44199), Vietnam: Vinh Phuc Province: Tam Dao National Park (21°30.36'N, 105°33.49'E, ca. 440 m), 16.VII.2007, D.S. Pham leg. ***Paratypes*** 3♀ (IZCAS-Ar44200–44202), same data as holotype; 1♀ (IZCAS-Ar44203), Tam Dao National Park (21°26.27'N, 105°37.41'E, ca. 310 m), 14.IV.2007, same collector; 1♂1♀ (IZCAS-Ar44204–44205), Tam Dao National Park (21°31.57'N, 105°33.15'E, ca. 1010 m), 21.VIII.2007, same collector; 1♂ (IZCAS-Ar44206), Tam Dao National Park (21°31.53'N, 105°32.37'E, ca. 500 m), 18.X.2007, same collector.

#### Etymology.

The specific name is a patronym in honor of Prof. Wayne P. Maddison (Vancouver, Canada), a leading arachnologist in jumping spiders, who has made significant contributions to the taxonomy of salticids worldwide; noun (name) in genitive case.

#### Diagnosis.

**Figure 7.**
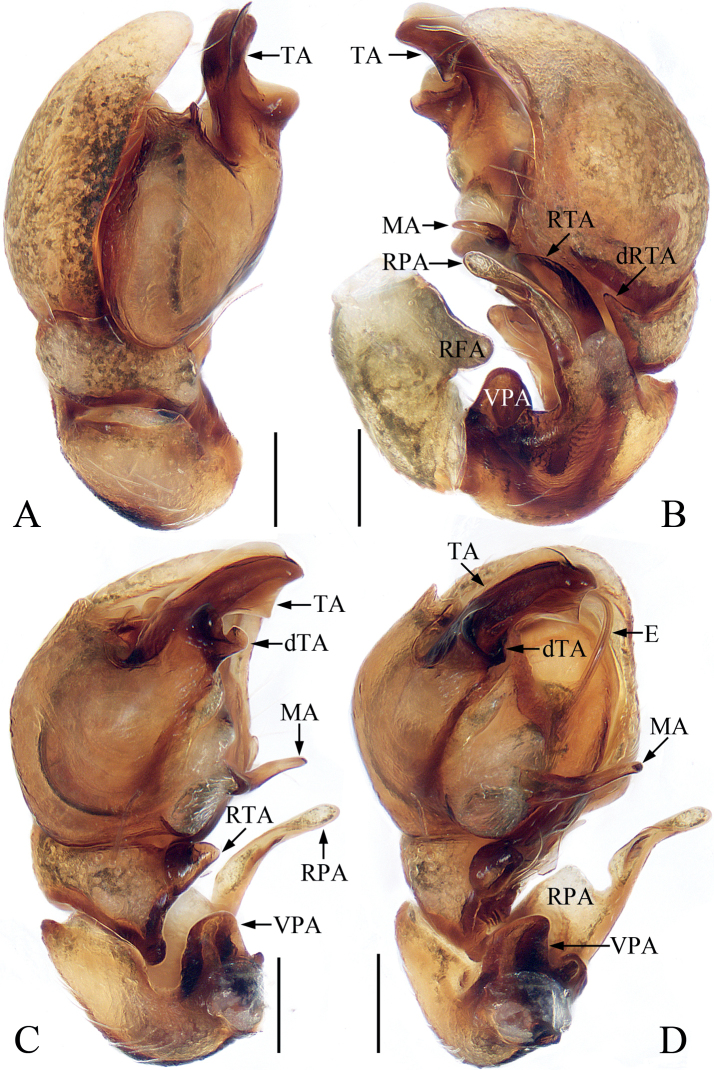
Male palp of *Eupoamaddisoni* sp. nov., holotype **A** prolateral **B** retrolateral **C** ventral **D** ventro-retrolateral. Scale bars: 0.1 mm. Abbreviations: dTA – division of terminal apophysis; dRTA – dorso-retrolateral tibial apophysis; E – embolus; MA – median apophysis; RFA – retrolateral femoral apophysis; RPA – retrolateral patellar apophysis; RTA – retrolateral tibial apophysis; TA – terminal apophysis; VPA – ventral patellar apophysis.

**Figure 8.**
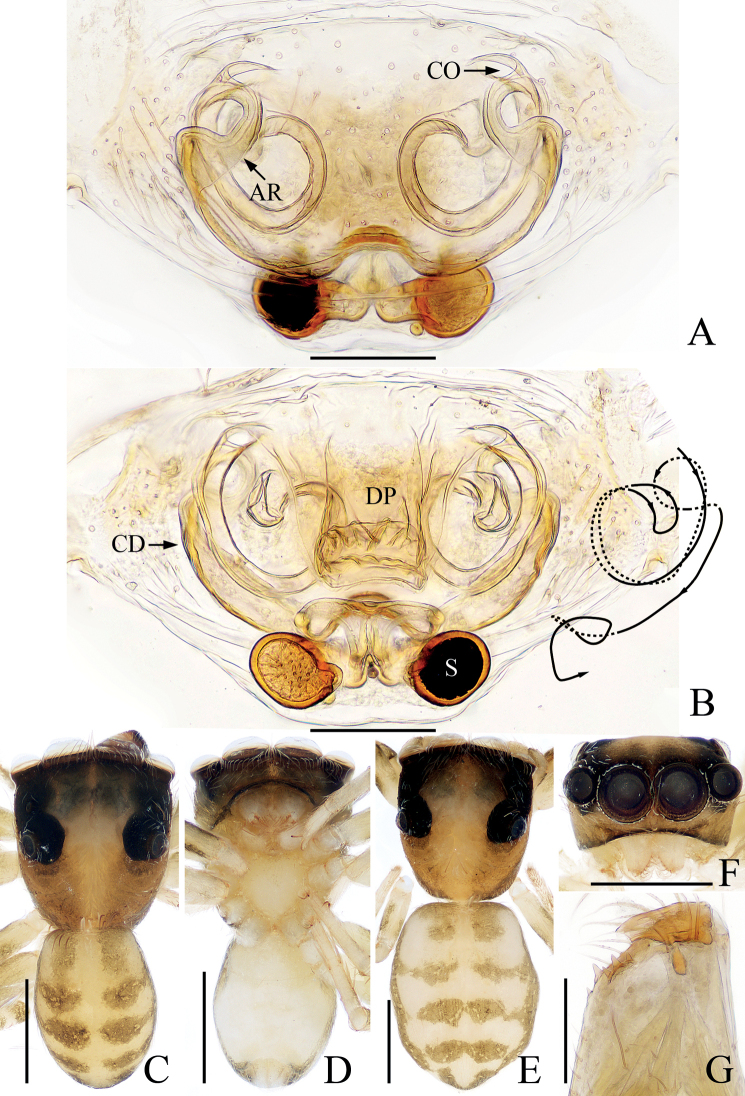
*Eupoamaddisoni* sp. nov., male holotype and female paratype **A** epigyne, ventral **B** vulva, dorsal **C** holotype habitus, dorsal **D** ditto, ventral **E** female paratype habitus, dorsal **F** holotype carapace, frontal **G** holotype chelicera, posterior. Scale bars: 0.1 mm (**A, B, G**); 0.5 mm (**C–F**). Abbreviations: AR – atrial ridge; CD – copulatory duct; CO – copulatory opening; DP – dorsal epigynal plate; S – spermatheca.

The male of *Eupoamaddisoni* sp. nov. closely resembles *E.nezha* Maddison & Zhang, 2007 in having a similar palp, especially the distally bifurcated RPA, but it can be easily distinguished by the following: (1) the shorter ramus of the RPA is ~ 1/2 the longer ramus length in retrolateral view (Fig. 7B), whereas it is < 1/10 of the longer ramus length in *E.nezha* (Maddison et al. 2007: fig. 2); (2) the TA has a curved dTA (Fig. 7C, D), whereas this is absent in *E.nezha* (Maddison et al. 2007: figs 1, 3). The female of this new species resembles *E.prima* Żabka, 1985 in having similar habitus markings and a similar epigyne, but it can be easily distinguished by the spermathecae, which are oval and separated from each other by approximately their width (Fig. 8B), whereas they are almost pyriform and touching in *E.prima* (Żabka 1985: fig. 169).

#### Description.

**Male** (Figs 7, 8C, D, F, G). Total length 1.60. Carapace 0.86 long 0.78 wide. Abdomen 0.78 long, 0.60 wide. Clypeus 0.05 high. Eye sizes and inter-distances: AME 0.24, ALE 0.16, PLE 0.14, AERW 0.79, PERW 0.73, EFL 0.49. Legs: I 1.36 (0.43, 0.20, 0.30, 0.25, 0.18), II 1.27 (0.40, 0.18, 0.28, 0.23, 0.18), III 1.24 (0.38, 0.18, 0.25, 0.25, 0.18), IV 1.69 (0.60, 0.23, 0.33, 0.33, 0.20). Carapace pale to dark yellow except bilateral sides of eye field, and posterior eyes bases dark, covered with white and pale setae, with pair of indistinct dark patches medially on eye field, and tapered, longitudinal, sub-triangular patch extending from the middle of PMEs to posterior margin; fovea indistinct. Chelicerae pale, with two promarginal and three retromarginal teeth. Endites sub-square. Labium darker than chelicerae and endites. Sternum almost heart-shaped, with straight anterior margin. Legs pale to green-brown, with three pairs of ventral spines on metatarsi I. Abdomen elongated, dorsum yellow to dark brown, with pair of longitudinal brown patches anteriorly followed by three pairs of transverse, dark brown patches, covered entirely by scutum; venter pale, without distinct markings. Palp (Fig. 7A–D): femur longer than wide, with blunt, sub-triangular retrolateral apophysis; patella sclerotized, with sub-trapeziform ventral apophysis, and distally bifurcated retrolateral apophysis acutely narrowed anteromedially, and with shorter ramus ~ 1/2 the longer ramus length; tibia short, with flat, irregular retrolateral apophysis and tapered, apically pointed dorso-retrolateral apophysis; bulb sub-oval; MA elongated, forming small hook at distal end; TA complex, with sclerotized, curved, basal division; embolus slender, flagelliform, partly visible.

**Female** (Fig. 8A, B, E). Total length 2.05. Carapace 0.91 long 0.83 wide. Abdomen 1.15 long, 0.91 wide. Clypeus 0.06 high. Eye sizes and inter-distances: AME 0.25, ALE 0.17, PLE 0.15, AERW 0.83, PERW 0.80, EFL 0.51. Legs: I 1.83 (0.55, 0.30, 0.40, 0.38, 0.20), II 1.52 (0.45, 0.23, 0.30, 0.34, 0.20), III 1.49 (0.45, 0.20, 0.30, 0.34, 0.20), IV 2.09 (0.70, 0.25, 0.52, 0.40, 0.22). Habitus (Fig. 8E) similar to that of male except without dorsal abdominal scutum and with three pairs of ventral spines on tibiae I. Epigyne (Fig. 8A, B): slightly wider than long, with large, irregular atrium and rectangular dorsal plate; copulatory openings located anteriorly, with C-shaped margins, separated from each other by ~ 3.5× the spermathecal width; copulatory ducts long, following a complex path; spermathecae oval, separated from each other ~ 3/4 of their width.

#### Distribution.

Known only from the type locality in Vinh Phuc Province, Vietnam.

### ﻿ Eupoa ninhbinh sp. nov.

Taxon classificationAnimaliaAraneaeSalticidae

48E06353-BE9F-5A44-8D51-BE59D75E9F70

https://zoobank.org/CDB9C82D-3D10-4841-9474-938CFEDD0AF9

Figs 9 , 10

#### Type material.

***Holotype*** ♂ (IZCAS-Ar44207), Vietnam: Ninh Binh Province: Cuc Phuong National Park, 1–30.XII.2007, D.S. Pham leg. ***Paratype*** 1♂ (IZCAS-Ar44208), Cuc Phuong National Park, 1–30.VII.2007, D.S. Pham leg.

#### Etymology.

The species is named after the type locality; noun in apposition.

#### Diagnosis.

**Figure 9.**
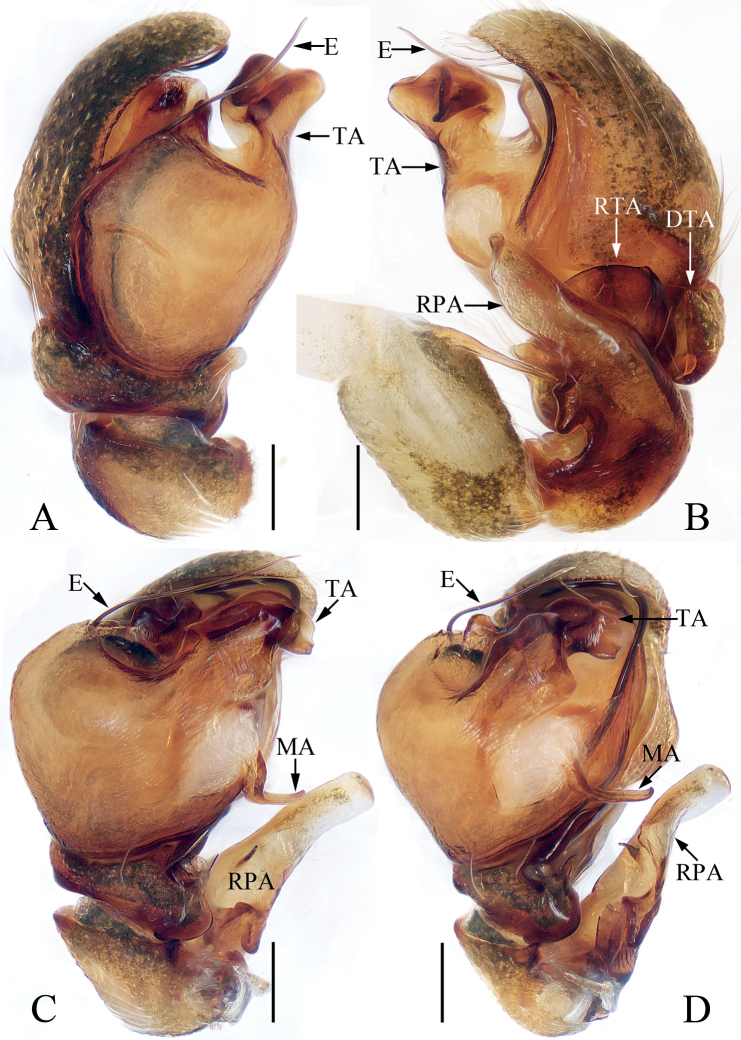
Male palp of *Eupoaninhbinh* sp. nov., holotype **A** prolateral **B** retrolateral **C** ventral **D** ventro-retrolateral. Scale bars: 0.1 mm. Abbreviations: DTA – dorsal tibial apophysis; E – embolus; MA – median apophysis; RPA – retrolateral patellar apophysis; RTA – retrolateral tibial apophysis; TA – terminal apophysis.

*Eupoaninhbinh* sp. nov. can be easily distinguished from other congeners by the presence of a large, baso-retrolateral femoral spine on the male palp (Fig. 9B), which is absent in all others (Metzner 2023).

#### Description.

**Figure 10.**
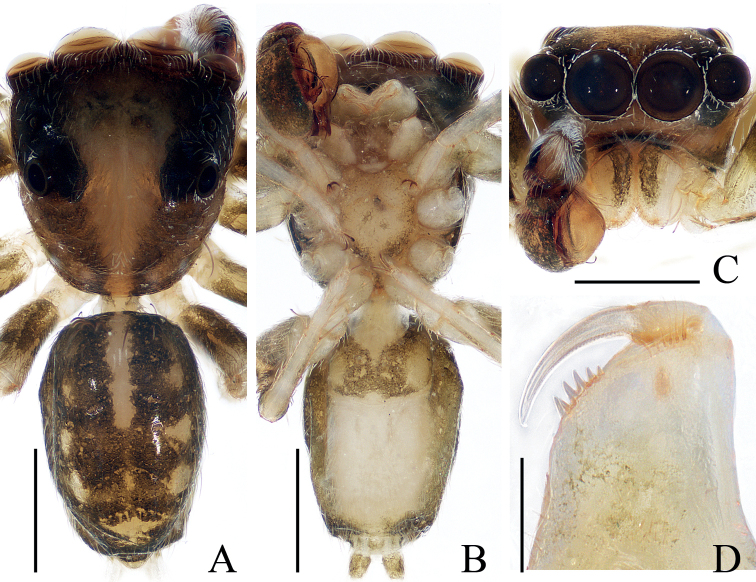
*Eupoaninhbinh* sp. nov., holotype **A** habitus, dorsal **B** ditto, ventral **C** carapace, frontal **D** chelicera, posterior. Scale bars: 0.1 mm (**D**); 0.5 mm (**A–C**).

**Male** (Figs 9, 10). Total length 2.01. Carapace 0.97 long 0.92 wide. Abdomen 0.99 long, 0.67 wide. Clypeus 0.07 high. Eye sizes and inter-distances: AME 0.28, ALE 0.19, PLE 0.16, AERW 0.95, PERW 0.80, EFL 0.55. Legs: I 1.72 (0.53, 0.28, 0.40, 0.33, 0.18), II 1.42 (0.43, 0.25, 0.28, 0.28, 0.18), III 1.47 (0.43, 0.23, 0.28, 0.35, 0.18), IV 2.04 (0.68, 0.28, 0.48, 0.40, 0.20). Carapace yellow to dark brown, with indistinct patch medially on eye field, and tapered, central, longitudinal, yellow patch extending across thorax; fovea indistinct. Chelicerae pale, with one or two promarginal and five retromarginal teeth. Endites, labium, sternum colored as chelicerae. Legs pale to green-brown, with one and three pairs of ventral spines on tibiae I and metatarsi I, respectively. Abdomen elongated, dorsum dark brown, with longitudinal, central stripe anteriorly, three pairs of yellow spots laterally, and quadrangular yellow patch posteriorly, covered entirely by scutum; venter pale. Palp (Fig. 9A–D): femur enlarged, with apically pointed baso-retrolateral spine more than half its length; patella sclerotized, with flat, broad retrolateral apophysis bearing sub-trapeziform division at base of posterior margin, and spine-shaped inner division; tibia short, with flat retrolateral apophysis and short, tapered dorsal apophysis with slightly pointed tip; bulb swollen, almost oval; MA slender, membranous at base, curved medially, forming hook at distal end; TA well-developed, irregularly-shaped; embolus slender, flagelliform.

**Female.** Unknown.

#### Distribution.

Known only from the type locality in Ninh Binh Province, Vietnam.

#### Comments.

The species is only known from the male, so there is a possibility that it is conspecific with one of two described species (*Eupoadaklak* Logunov & Marusik, 2014 and *E.hainanensis* Peng & Kim, 1997) that are also known only from females. However, *E.ninhbinh* sp. nov. has specific habitus markings (see description) that differ from these two species.

### ﻿ Eupoa zabkai sp. nov.

Taxon classificationAnimaliaAraneaeSalticidae

4AFD30FC-E47D-502D-9AB1-EC700CA8F203

https://zoobank.org/F0FE9234-552F-49EA-8E5A-119D3906FC4D

Figs 11 , 12

#### Type material.

***Holotype*** ♂ (IZCAS-Ar44209), Vietnam: Vinh Phuc Province: Tam Dao National Park (21°30.52'N, 105°34.26'E, ca. 510 m), 19.IX.2007, D.S. Pham leg. ***Paratypes*** 1♀ (IZCAS-Ar44210), same data as holotype; 1♀ (IZCAS-Ar44211), Tam Dao National Park (21°29.43'N, 105°37.01'E, ca. 1080 m), 12.IV.2007, same collector; 2♂ (IZCAS-Ar44212–44213), Tam Dao National Park (21°31.25'N, 105°33.50'E, ca. 690 m), 13.VI.2007, same collector; 2♀ (IZCAS-Ar44214–44215), Tam Dao National Park (21°29.03'N, 105°37.19'E, ca. 790 m), 18.VII.2007, same collector; 1♀ (IZCAS-Ar44216), Tam Dao National Park (21°29.23'N, 105°37.20'E, ca. 870 m), 19.VII.2007, same collector; 1♀ (IZCAS-Ar44217), Tam Dao National Park (21°28.34'N, 105°38.09'E, ca. 1010 m), 22.VIII.2007, same collector; 1♀ (IZCAS-Ar44218), Tam Dao National Park (21°29.03'N, 105°37.19'E, ca. 790 m), 17.X.2007, same collector; 1♀ (IZCAS-Ar44219), Tam Dao National Park (21°30.52'N, 105°34.26'E, ca. 510 m), 13.XI.2007, same collector.

#### Etymology.

The species name is a patronym in honor of Prof. Marek Żabka (Siedlce, Poland), a leading arachnologist in jumping spiders, who contributed significantly to the taxonomy of jumping spiders from Vietnam; noun (name) in genitive case.

#### Diagnosis.

**Figure 11.**
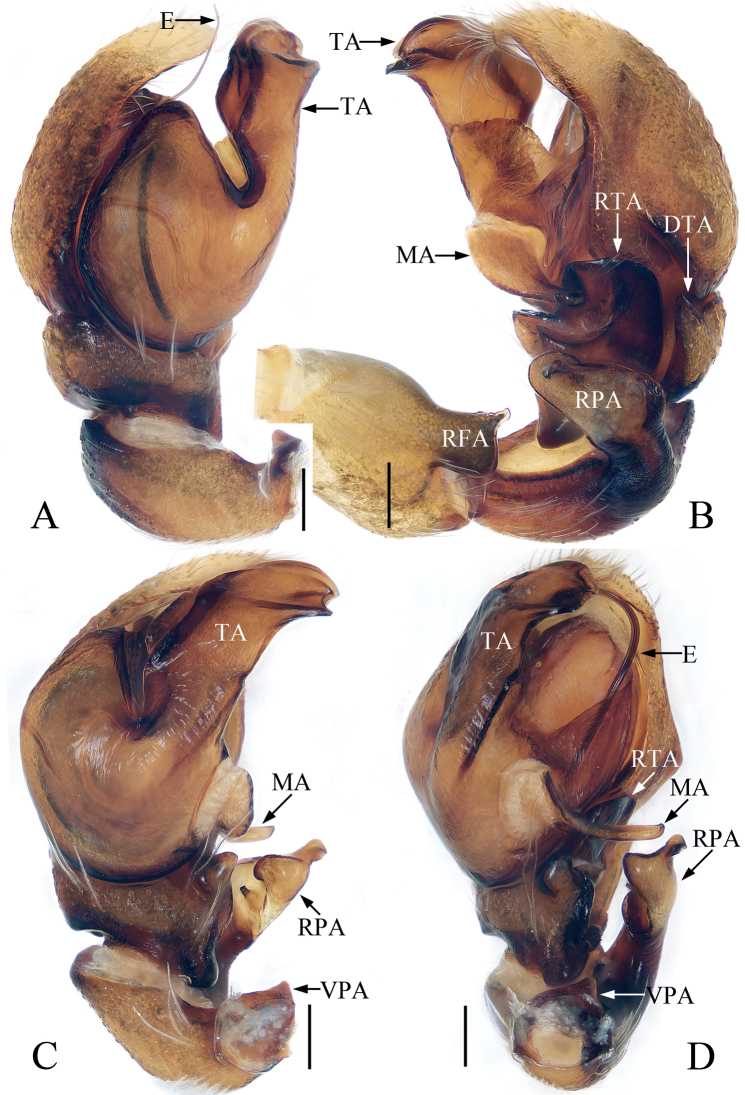
Male palp of *Eupoazabkai* sp. nov., holotype **A** prolateral **B** retrolateral **C** ventral **D** ventro-retrolateral. Scale bars: 0.1 mm. Abbreviations: DTA – dorsal tibial apophysis; E – embolus; MA – median apophysis; RFA – retrolateral femoral apophysis; RPA – retrolateral patellar apophysis; RTA – retrolateral tibial apophysis; TA – terminal apophysis; VPA – ventral patellar apophysis.

**Figure 12.**
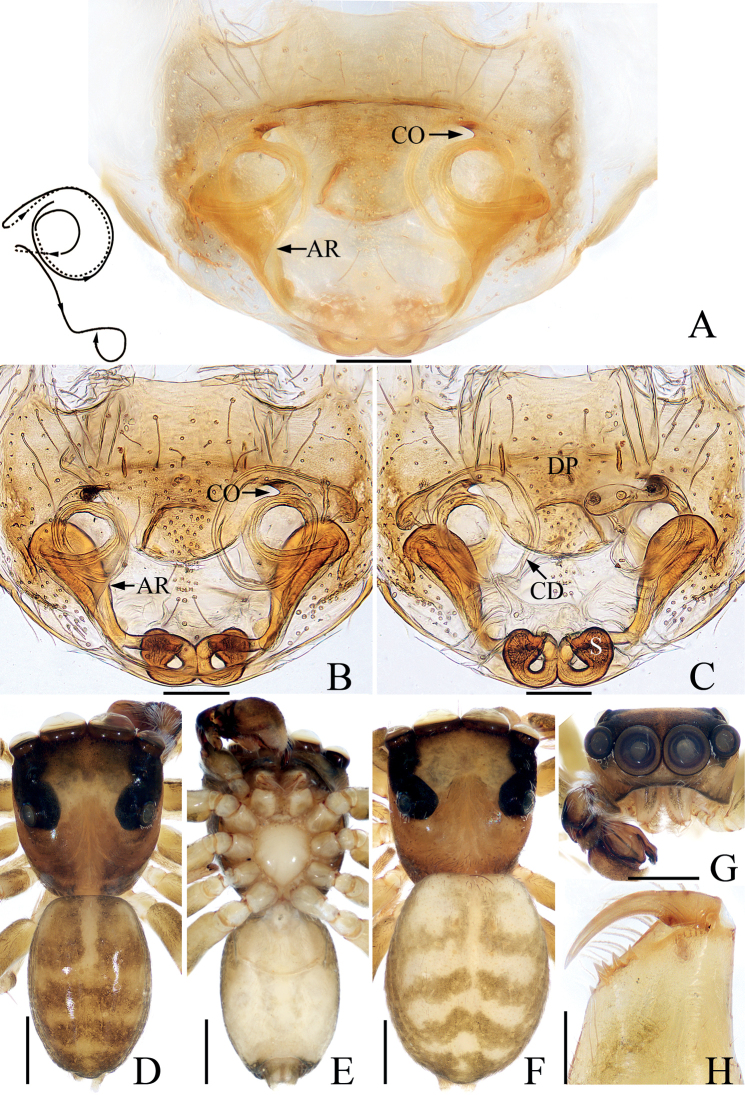
*Eupoazabkai* sp. nov., male holotype and female paratype **A, B** epigyne, ventral **C** vulva, dorsal **D** holotype habitus, dorsal **E** ditto, ventral **F** female paratype habitus, dorsal **G** holotype carapace, frontal **H** holotype chelicera, posterior. Scale bars: 0.1 mm (**A–C, H**); 0.5 mm (**D–G**). Abbreviations: AR – atrial ridge; CD – copulatory duct; CO – copulatory opening; DP – dorsal epigynal plate; S – spermatheca.

The male of *Eupoazabkai* sp. nov. resembles *E.jingwei* Maddison & Zhang, 2007 in the general shape of palp, but it can easily be distinguished by the presence of an RFA (Fig. 11B), whereas it is absent in *E.jingwei* (Maddison et al. 2007: fig. 9). The species also resembles *E.prima* Żabka, 1985, but it differs in: (1) the RPA being shorter than the patella in retrolateral view and with a short, inner division (Fig. 11B–D), but the RPA is almost 1.5× longer than the patella and lacks an inner division in *E.prima* (Żabka 1985: figs 161–163); (2) the epigyne has a dorsal plate (Fig. 12B, C), whereas this is indistinct in *E.prima* (Żabka 1985: fig. 169); (3) the copulatory ducts are connected to the base of the spermathecae (Fig. 12B, C), whereas in *E.prima*, they are lateral to the spermathecae (Żabka 1985: fig. 169).

#### Description.

**Male** (Figs 11, 12D, E, G, H). Total length 2.87. Carapace 1.37 long 1.26 wide. Abdomen 1.53 long, 1.03 wide. Clypeus 0.09 high. Eye sizes and inter-distances: AME 0.41, ALE 0.28, PLE 0.16, AERW 1.31, PERW 1.15, EFL 0.76. Legs: I 2.37 (0.73, 0.38, 0.55, 0.48, 0.23), II 2.07 (0.63, 0.33, 0.45, 0.43, 0.23), III 2.02 (0.58, 0.30, 0.43, 0.48, 0.23), IV 2.99 (0.93, 0.38, 0.80, 0.63, 0.25). Carapace elevated, pale to brown except bilateral sides of eye field and posterior eyes bases dark; fovea indistinct. Chelicerae yellow, with two promarginal and four retromarginal teeth. Endites, labium and sternum paler than chelicerae. Legs pale to yellow, with one ventral spine on tibiae I and three pairs of ventral spines on metatarsi I. Abdomen elongate-oval, dorsum yellow to brown, with three longitudinal stripes anteriorly, followed by alternating transverse brown and yellow stripes, covered entirely by scutum; venter pale. Palp (Fig. 11A–D): femur enlarged, with sub-square retrolateral apophysis bearing finger-shaped antero-marginal division; patella sclerotized, longer than wide; RPA well-developed, broadened medially, with irregular distal division and short, bar-shaped inner division; tibia short, with flat, broad, irregularly-shaped retrolateral apophysis and triangular dorsal apophysis with pointed tip; bulb swollen, almost oval; MA weakly sclerotized at base, slightly curved medially, hooked distally; TA well-developed, irregularly-shaped, > 4× longer than wide, forming ridge distally; embolus long, flagelliform.

**Female** (Fig. 12A–C, F). Total length 2.81. Carapace 1.29 long 1.17 wide. Abdomen 1.67 long, 1.29 wide. Clypeus 0.08 high. Eye sizes and inter-distances: AME 0.38, ALE 0.26, PLE 0.21, AERW 1.22, PERW 1.08, EFL 0.69. Legs: I 2.57 (0.78, 0.43, 0.63, 0.50, 0.23), II 2.15 (0.68, 0.33, 0.48, 0.43, 0.23), III 2.19 (0.68, 0.33, 0.45, 0.50, 0.23), IV 3.34 (1.10, 0.43, 0.88, 0.68, 0.25). Habitus (Fig. 12F) similar to that of male except without dorsal abdominal scutum, and with three pairs of ventral spines on tibiae I. Epigyne (Fig. 12A–C): slightly wider than long, with pair of antero-marginal depressions, large, irregular atrium, and linguiform dorsal plate; copulatory openings anteriorly located, separated from each other by more than atrial width, with C-shaped margins; copulatory ducts long, forming complex coils, connected to base of spermathecae; spermathecae sub-oval, separated from each other by ~ 2/3 of their width.

#### Distribution.

Known only from the type locality in Vinh Phuc Province, Vietnam.

### ﻿Genus Indopadilla

Taxon classificationAnimaliaAraneaeSalticidae

Caleb & Sankaran, 2019

23262EC4-3A99-5C05-A120-C1C8AB844FFC

#### Type species.

*Indopadilladarjeeling* Caleb & Sankaran, 2019 from India.

#### Comments.

*Indopadilla* is placed in the tribe Baviini with four other genera and is represented by 14 species distributed in tropical Asia (Maddison et al. 2020; WSC 2023). The genus is rather well studied, but more than half (8) of the species are known only from a single sex, indicating it needs further attention (WSC 2023). To date, only one species has been recorded from Vietnam.

### ﻿ Indopadilla cuc sp. nov.

Taxon classificationAnimaliaAraneaeSalticidae

E6C106A6-F28D-594D-91F3-CDAAF728E94C

https://zoobank.org/80587BFF-739C-4B8E-8658-DA569E9705B4

Figs 13 , 14

#### Type material.

***Holotype*** ♂ (IZCAS-Ar44220), Vietnam: Ninh Binh Province: Cuc Phuong National Park, Disturbed Forest (20°15.30'N, 105°42.55'E, ca. 250 m), 18.VIII.2007, D.S. Pham leg. ***Paratypes*** 1♀ (IZCAS-Ar44221), same data as holotype; 1♂ (IZCAS-Ar44222), Cuc Phuong National Park (20°21.44'N, 105°34.21'E, ca. 410 m), 2.IV.2007, same collector; 1♀ (IZCAS-Ar44223), same site and collector as holotype, 3.VI.2007; 1♀ (IZCAS-Ar44224), Cuc Phuong National Park (20°21.22'N, 105°37.03'E, ca. 440 m), 5.VI.2007, same collector; 1♀ (IZCAS-Ar44225), Cuc Phuong National Park (20°20.23'N, 105°36.23'E, ca. 390 m), 5.VII.2007, same collector; 1♂ (IZCAS-Ar44226), Cuc Phuong National Park (20°19.45'N, 105°37.30'E, ca. 270 m), 20.VII.2008, S. Li leg.

#### Etymology.

The species is named after the type locality; noun in apposition.

#### Diagnosis.

**Figure 13.**
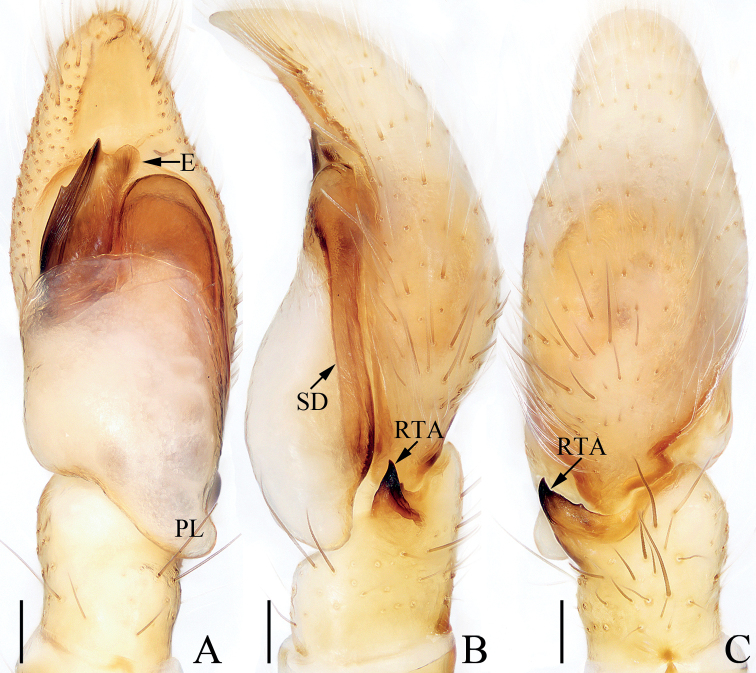
Male palp of *Indopadillacuc* sp. nov., holotype **A** ventral **B** retrolateral **C** dorsal. Scale bars: 0.1 mm. Abbreviations: E – embolus; PL – posterior lobe; RTA – retrolateral tibial apophysis; SD – sperm duct.

**Figure 14.**
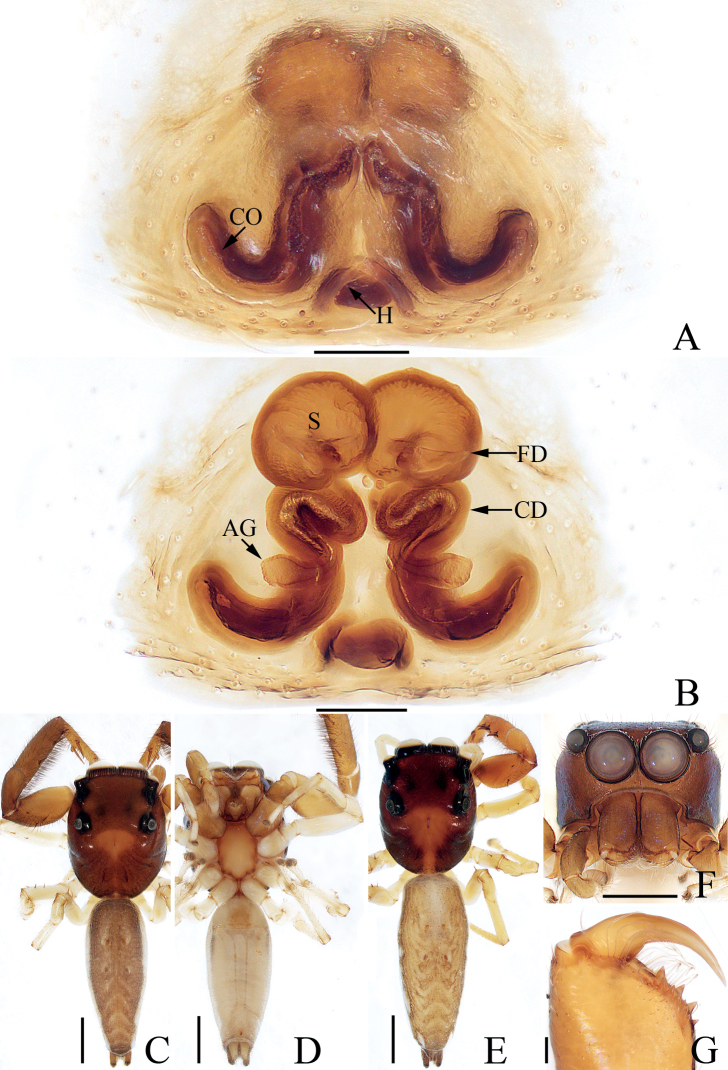
*Indopadillacuc* sp. nov., male holotype and female paratype **A** epigyne, ventral **B** vulva, dorsal **C** holotype habitus, dorsal **D** ditto, ventral **E** female paratype habitus, dorsal **F** holotype carapace, frontal **G** holotype chelicera, posterior. Scale bars: 0.1 mm (**A, B, G**); 1.0 mm (**C–F**). Abbreviations: AG – accessory gland; CD – copulatory duct; CO – copulatory opening; FD – fertilization duct; H – epigynal hood; S – spermatheca.

*Indopadillacuc* sp. nov. closely resembles *I.annamita* (Simon, 1903) in the habitus and copulatory organs, but it can be easily distinguished by the following: (1) the embolus is sheet-shaped and without an accompanying membranous division (Fig. 13A), whereas the embolus is spine-shaped and accompanied by a membranous division in *I.annamita* (Żabka 1988: fig. 46); (2) the RTA is tapered and < 1/3 the tibial length in retrolateral view (Fig. 13B), whereas the RTA is broadened anteromedially and > 1/2 the tibial length in *I.annamita* (Żabka 1988: fig. 47); (3) the epigyne has a posterior, half-round hood ~ 4/5 of the spermathecal width (Fig. 14A), whereas the epigyne has a broad hood > 2× the spermathecal width in *I.annamita* (Żabka 1988: fig. 50).

#### Description.

**Male** (Figs 13, 14C, D, F, G). Total length 6.15. Carapace 2.68 long 2.18 wide. Abdomen 3.42 long, 1.38 wide. Clypeus 0.09 high. Eye sizes and inter-distances: AME 0.71, ALE 0.34, PLE 0.32, AERW 1.88, PERW 1.85, EFL 1.26. Legs: I 7.96 (2.33, 1.25, 2.13, 1.55, 0.70), II 5.15 (1.60, 0.88, 1.18, 1.01, 0.48), III 4.78 (1.50, 0.75, 0.88, 1.15, 0.50), IV 5.88 (1.80, 0.75, 1.30, 1.53, 0.50). Carapace red-brown, with pair of dark spots medio-posteriorly on eye field, and fan-shaped yellow area anteromedially on thorax, covered with brown, thin setae; fovea red-brown, longitudinal, linear. Chelicerae yellow, with four promarginal and seven retromarginal teeth. Endites longer than wide, broadened distally. Labium darker than endites. Sternum ~ 1.5× longer than wide. Legs pale to brown, with dense setae ventrally on patellae I and tibiae I, three and two pairs of ventral spines on tibiae I and metatarsi I, respectively. Abdomen elongated, dorsum brown, with two pairs of muscle depressions anteromedially, and followed by four pale inverted V-shaped stripes; venter pale, with pair of longitudinal, dotted lines medially. Palp (Fig. 13A–C): tibia slightly longer than wide, with strongly sclerotized, tapered retrolateral apophysis ~ 1/3 tibial length, with pointed tip almost directed anteriorly in retrolateral view; cymbium longer than wide; bulb swollen medio-posteriorly, with blunt posterior lobe extending postero-retrolaterally; embolus broad, flat, originates from antero-prolateral portion of bulb, forming semi-circular notch at distal margin.

**Female** (Fig. 14A, B, E). Total length 6.32. Carapace 2.61 long 2.07 wide. Abdomen 3.75 long, 1.46 wide. Clypeus 0.09 high. Eye sizes and inter-distances: AME 0.71, ALE 0.33, PLE 0.32, AERW 1.88, PERW 1.82, EFL 1.25. Legs: I 5.43 (1.65, 1.03, 1.30, 0.95, 0.50), II 4.41 (1.30, 0.80, 1.05, 0.78, 0.48), III 3.99 (1.18, 0.68, 0.75, 0.90, 0.48), IV 5.28 (1.53, 0.75, 1.20, 1.30, 0.50). Habitus (Fig. 14E) similar to that of male except with darker carapace. Epigyne (Fig. 14A, B): slightly wider than long, with posterior, half-round hood close to epigastric furrow; copulatory openings elongated, slit-shaped, posterolaterally located; copulatory ducts strongly curved, with transversely extending accessory glands; spermathecae almost round, anteriorly located, partly overlapping; fertilization ducts originate from inner sides of posterior sub-margins of spermathecae, extending transversely.

#### Distribution.

Known only from the type locality in Ninh Binh Province, Vietnam.

### ﻿Genus Synagelides

Taxon classificationAnimaliaAraneaeSalticidae

Strand, 1906

7C7ACC09-51B8-58B4-93B0-AAB2CCA8D6F8

#### Type species.

*Synagelidesagoriformis* Strand, 1906 from Japan.

#### Comments.

The ant-like genus *Synagelides* is placed into the tribe Agoriini with the genus *Agorius* and is represented by 67 species mainly distributed in East and Southeast Asia (WSC 2023). The genus is unique for the male palp has a triangular femoral apophysis, an enlarged patella and a well-developed median apophysis retrolateral to the embolus. Most species of *Synagelides* can be further categorized into several groups according to their morphological features (Wang et al. 2020). The highest diversity of the genus occurs in China, but many undescribed species have also been found in our additional samples, indicating that the true diversity of the genus will increase. To date, only one species has been recorded from Vietnam.

### ﻿ Synagelides ani sp. nov.

Taxon classificationAnimaliaAraneaeSalticidae

0257E23F-3E79-591F-BB74-A3C1BA5EBABC

https://zoobank.org/2287FAE1-D406-4070-B8EA-6688BDF6951D

Figs 15 , 16

#### Type material.

***Holotype*** ♂ (IZCAS-Ar44227), Vietnam: Vinh Phuc Province: Tam Dao National Park (21°28.34'N, 105°38.09'E, ca. 1010 m), 13.VI.2007, D.S. Pham leg. ***Paratypes*** 1♂2♀ (IZCAS-Ar44228–44230), same data as holotype; 3♂2♀ (IZCAS-Ar44231–44235), Tam Dao National Park (21°27.53'N, 105°38.51'E, ca. 1080 m), 17.V.2007, same collector; 3♀ (IZCAS-Ar44236–44238), Tam Dao National Park (21°29.43'N, 105°37.01'E, ca. 1080 m), 19.VIII.2007, same collector; 1♀ (IZCAS-Ar44239), Tam Dao National Park (21°29.23'N, 105°37.23'E, ca. 870 m), 21.VIII.2007, same collector; 1♀ (IZCAS-Ar44240), Tam Dao National Park (21°30.52'N, 105°34.26'E, ca. 510 m), 17.X.2007, same collector; 2♀ (IZCAS-Ar44241–44242), Tam Dao National Park (21°28.34'N, 105°38.09'E, ca. 1010 m), 15.I.2008, same collector; 1♀ (IZCAS-Ar44243), Tam Dao National Park (21°27.53'N, 105°38.51'E, ca 1080 m), 12.III.2008, same collector.

#### Etymology.

The specific name is after one of the most popular surnames (An) in Vietnam; noun (name) in genitive case.

#### Diagnosis.

**Figure 15.**
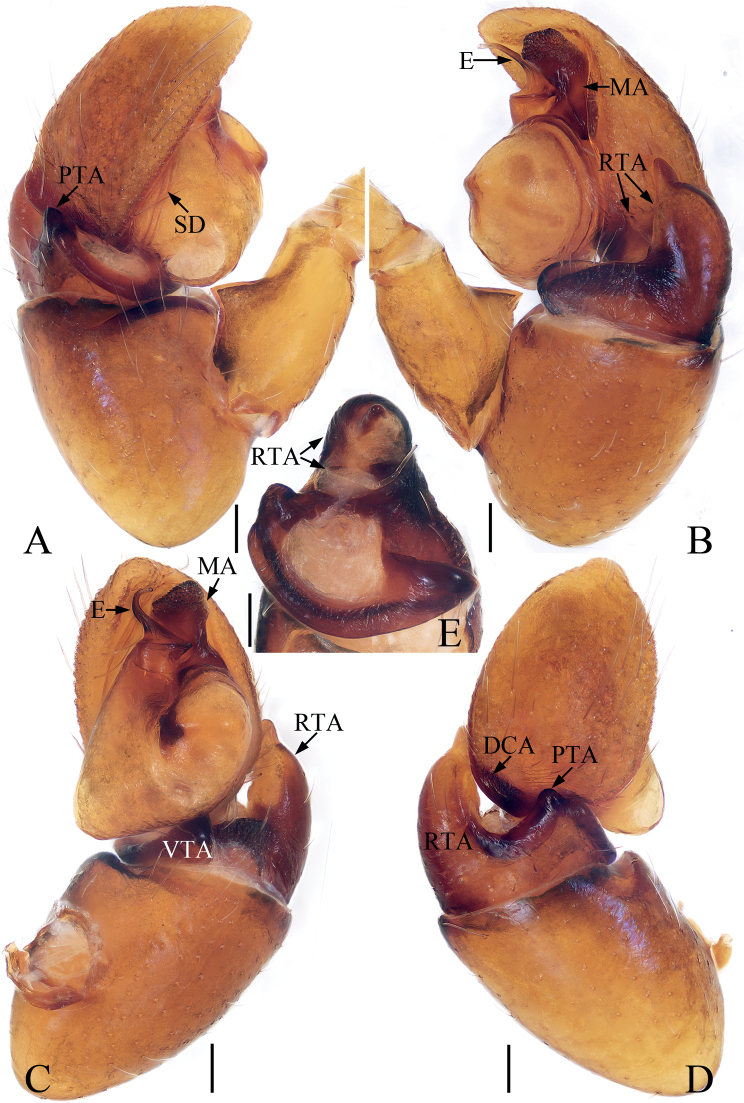
Male palp of *Synagelidesani* sp. nov., holotype and paratype **A** holotype, prolateral **B** ditto, retrolateral **C** ditto, ventral **D** ditto, dorsal **E** paratype tibia, prolatero-apical. Scale bars: 0.1 mm. Abbreviations: DCA – dorsal cymbial apophysis; E – embolus; MA – median apophysis; PTA – prolateral tibial apophysis; RTA – retrolateral tibial apophysis; SD – sperm duct; VTA – ventral tibial apophysis.

**Figure 16.**
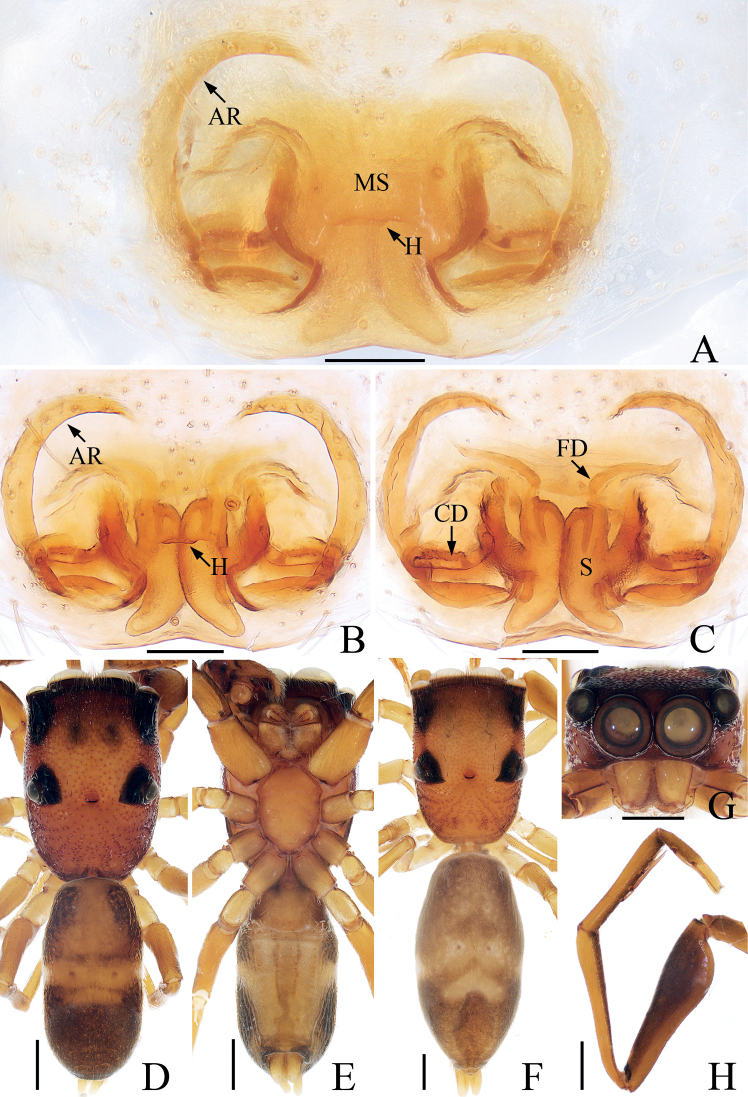
*Synagelidesani* sp. nov., male holotype and female paratype **A, B** epigyne, ventral **C** vulva, dorsal **D** holotype habitus, dorsal **E** ditto, ventral **F** female paratype habitus, dorsal **G** holotype carapace, frontal **H** holotype leg I, prolateral. Scale bars: 0.1 mm (**A–C**); 0.5 mm (**D–H**). Abbreviations: AR – atrial ridge; CD – copulatory duct; FD – fertilization duct; MS – median septum; H – epigynal hood; S – spermatheca.

The male of *Synagelidesani* sp. nov. closely resembles *S.leigongensis* Wang, Mi, Irfan & Peng, 2020 in having a very similar palp, especially the needle-shaped division of the RTA, but it can be easily distinguished by the RTA, which is acutely narrowed distally to a rather pointed tip and with a straight needle-shaped division in retrolateral view (Fig. 15B), but the RTA is blunt apically with a curved, needle-shaped division in *S.leigongensis* (Wang et al. 2020: fig. 5B). The female of this new species resembles *S.xingdouensis* Wang, Mi, Irfan & Peng, 2020 in having longitudinally extending spermathecae, but it can be easily distinguished by the following: (1) the presence of an epigynal hood (Fig. 16A, B), which is absent in *S.xingdouensis* (Wang et al. 2020: fig. 16A, B); (2) the atrium is as wide as the epigyne (Fig. 16A, B), whereas it is ~ 1/3 the epigynal width in *S.xingdouensis* (Wang et al. 2020: fig. 16A, B).

#### Description.

**Male** (Figs 15, 16D, E, G, H). Total length 3.94. Carapace 1.98 long 1.40 wide. Abdomen 2.04 long, 1.02 wide. Clypeus 0.02 high. Eye sizes and inter-distances: AME 0.48, ALE 0.26, PLE 0.26, AERW 1.35, PERW 1.33, EFL 1.07. Legs: I 5.94 (1.88, 1.75, 1.33, 0.63, 0.35), II 3.40 (1.01, 0.50, 0.83, 0.73, 0.33), III 3.46 (0.98, 0.50, 0.80, 0.85, 0.33), IV 4.53 (1.25, 0.65, 1.20, 1.08, 0.35). Carapace stippled, yellow-red to dark, covered with white and brown setae, with pair of irregular dark patches anteromedially on eye field; fovea oval. Chelicerae yellow, with two promarginal teeth and one retromarginal fissidental tooth with two cusps. Endites red-yellow, almost square, bearing dense setae on paler inner margins. Labium colored as endites, almost linguiform. Sternum red-yellow, longer than wide, almost shield-shaped. Legs yellow except femora I red-brown, with seven (2-2-2-1) and two spines on tibiae I and metatarsi I, respectively. Abdomen elongated, dorsum yellow to dark brown, with longitudinal, sub-trapeziform patch anteriorly, pair of muscle depressions, two transverse, yellow stripes medially, and two clusters of white setae mediolaterally; venter yellow to brown, with dotted lines medially. Palp (Fig. 15A–E): femur longer than wide, with triangular disto-prolateral apophysis; patella enlarged, longer than wide; tibia short, with sclerotized, sub-triangular PTA, and robust RTA with dolphin-shaped longitudinal part, and membranous, needle-shaped inner division; cymbium longer than wide in ventral view; bulb swollen; embolus twisted, tapered; MA sclerotized, irregular, retrolateral to embolus, with small processes distally.

**Female** (Fig. 16A–C, F). Total length 5.39. Carapace 2.29 long 1.61 wide. Abdomen 3.14 long, 1.57 wide. Clypeus 0.02 high. Eye sizes and inter-distances: AME 0.53, ALE 0.31, PLE 0.31, AERW 1.64, PERW 1.63, EFL 1.39. Legs: I 5.14 (1.63, 1.50, 1.18, 0.50, 0.33), II 3.67 (1.08, 0.55, 0.88, 0.78, 0.38), III 3.92 (1.10, 0.55, 0.88, 1.01, 0.38), IV 5.28 (1.38, 0.75, 1.40, 1.35, 0.40). Habitus (Fig. 16F) similar to that of male except paler and without distinct dark patches on eye field. Epigyne (Fig. 16A–C): wider than long, with small, broad, central hood; atrium oval, separated by the broad, irregularly-shaped median septum, with pair of arc-shaped lateral ridges; copulatory openings beneath the base of atrial ridges; copulatory ducts straight, transversely extending at proximal half, continuing, slightly curving and followed by invisible parts; spermathecae longitudinal extending, ~ 2.5× longer than wide, touched, tapered posteriorly; fertilization ducts originate from anterior portions of spermathecae, lamellar.

#### Distribution.

Known only from the type locality in Vinh Phuc, Vietnam.

### ﻿ Synagelides mii sp. nov.

Taxon classificationAnimaliaAraneaeSalticidae

3A73FAEB-539D-5DD2-A14B-EFED9E66BBC2

https://zoobank.org/6529AA87-36A3-4706-8D01-E5C861A054D3

Figs 17 , 18

#### Type material.

***Holotype*** ♂ (IZCAS-Ar44244), Vietnam: Ninh Binh Province: Cuc Phuong National Park, Disturbed Forest (20°16.07'N, 105°42.04'E, ca. 250 m), 18.VIII.2007, D.S. Pham leg. ***Paratypes*** 1♂1♀ (IZCAS-Ar44245–44246), same data as holotype; 1♂ (IZCAS-Ar44247), Cuc Phuong National Park, 1–30.IV.2007, same collector; 1♂ (IZCAS-Ar44248), Cuc Phuong National Park (20°15.30'N, 105°42.55'E, ca. 250 m), 8.V.2007, same collector; 3♂3♀ (IZCAS-Ar44249–44254), Cuc Phuong National Park (20°19.58'N, 105°37.38'E, ca. 300 m), 3.VII.2007, same collector; 3♀ (IZCAS-Ar44255–44257), Cuc Phuong National Park (20°17.07'N, 105°40.25'E, ca. 270 m), 6.VII.2007, same collector; 1♀ (IZCAS-Ar44258), Cuc Phuong National Park (20°17.07'N, 105°40.25'E, ca. 270 m), 9.X.2007, same collector; 1♀ (IZCAS-Ar44259), Cuc Phuong National Park (20°16.07'N, 105°42.04'E, ca. 250 m), 6.XI.2007, same collector; 1♂1♀ (IZCAS-Ar44260–44261), Cuc Phuong National Park (20°23.16'N, 105°32.03'E, ca. 200 m), 7.XI.2007, same collector; 1♂ (IZCAS-Ar44262), Cuc Phuong National Park (20°22.27'N, 105°33.05'E, ca. 380 m), 8.I.2008, same collector; 2♂ (IZCAS-Ar44263–44264), Cuc Phuong National Park (20°15.30'N, 105°42.55'E, ca. 250 m), 6.II.2008, same collector.

#### Etymology.

The specific name is a patronym after Prof. Xiaoqi Mi (Tongren, China), who help us greatly with this taxonomic study; noun (name) in genitive case.

#### Diagnosis.

**Figure 17.**
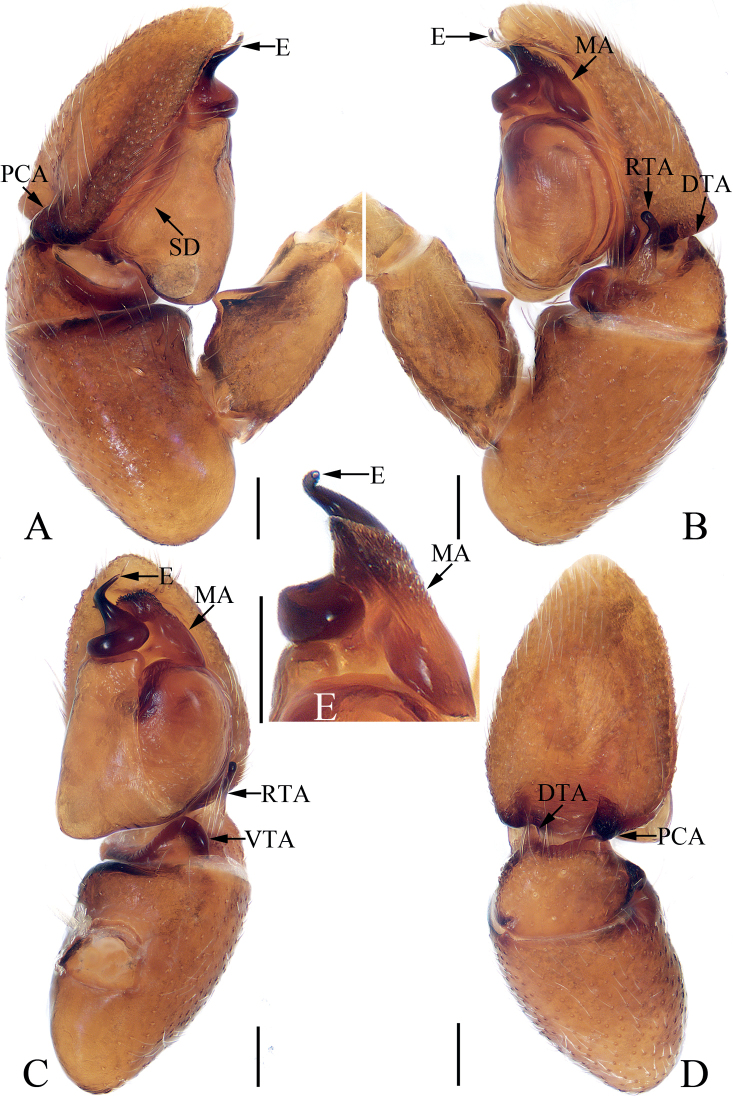
Male palp of *Synagelidesmii* sp. nov., holotype and paratype **A** holotype, prolateral **B** ditto, retrolateral **C** ditto, ventral **D** ditto, dorsal **E** paratype embolus and median apophysis, retrolateral. Scale bars: 0.1 mm. Abbreviations: DTA – dorsal tibial apophysis; E – embolus; MA – median apophysis; PCA – prolateral cymbial apophysis; RTA – retrolateral tibial apophysis; SD – sperm duct; VTA – ventral tibial apophysis.

**Figure 18.**
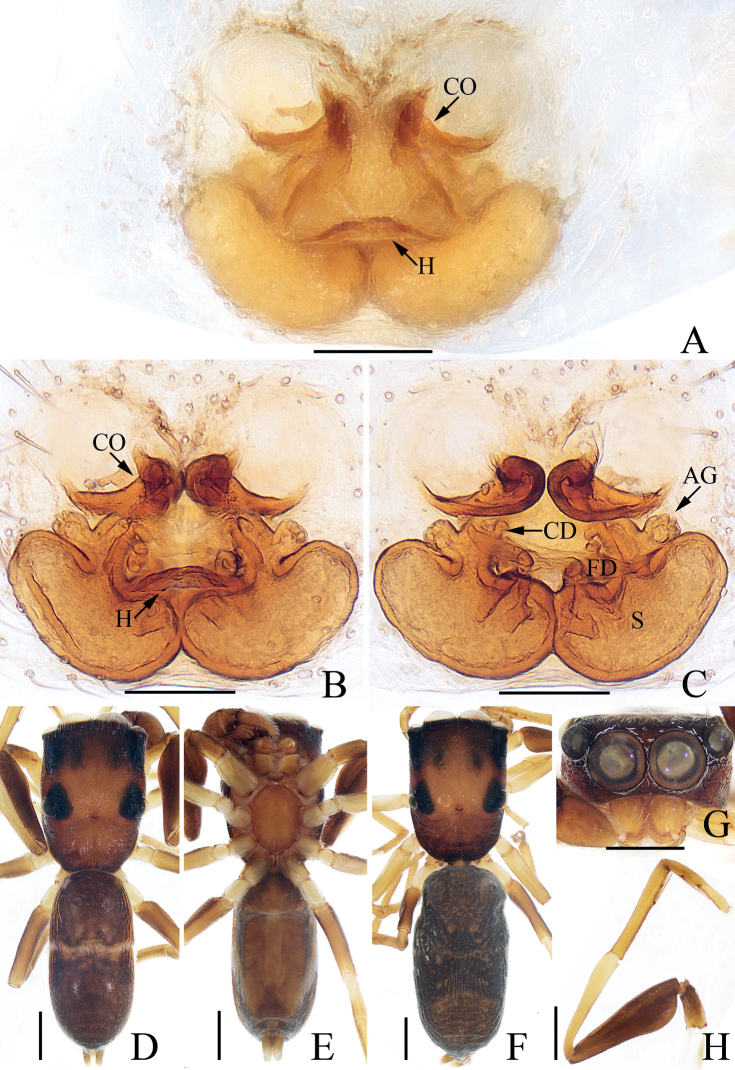
*Synagelidesmii* sp. nov., male holotype and female paratype **A, B** epigyne, ventral **C** vulva, dorsal **D** holotype habitus, dorsal **E** ditto, ventral **F** female paratype habitus, dorsal **G** holotype carapace, frontal **H** holotype leg I, prolateral. Scale bars: 0.1 mm (**A–C**); 0.5 mm (**D–H**). Abbreviations: AG – accessory gland; CD – copulatory duct; CO – copulatory opening; FD – fertilization duct; H – epigynal hood; S – spermatheca.

The male of *Synagelidesmii* sp. nov. resembles that of *S.tangi* Liu, Chen, Xiao, Xu & Peng, 2017 in having a similar RTA, but it can be easily distinguished by the presence of a DTA and by the blunt PCA (Fig. 17A, B), whereas a DTA is absent and the PCA is pointed in *S.tangi* (Liu et al. 2017: figs 5G, 6D). The female of this new species resembles that of *S.leigongensis* Wang, Mi, Irfan & Peng, 2020 in having very large, transversely extending spermathecae, but it can be easily distinguished by the following: (1) the presence of an epigynal hood (Fig. 18A, B), which is absent in *S.leigongensis* (Wang et al. 2020: fig. 6A); (2) the spermathecae extend anterolaterally (Fig. 18B, C), whereas they extend posterolaterally in *S.leigongensis* (Wang et al. 2020: fig. 6C).

#### Description.

**Male** (Figs 17, 18D, E, G, H). Total length 3.31. Carapace 1.54 long 1.08 wide. Abdomen 1.83 long, 0.88 wide. Clypeus 0.02 high. Eye sizes and inter-distances: AME 0.38, ALE 0.22, PLE 0.20, AERW 1.08, PERW 1.08, EFL 0.92. Legs: I 4.07 (1.25, 1.10, 1.01, 0.43, 0.28), II 2.51 (0.70, 0.38, 0.60, 0.50, 0.33), III 2.64 (0.73, 0.38, 0.60, 0.60, 0.33), IV 3.39 (0.90, 0.43, 0.88, 0.83, 0.35). Carapace stippled, dark yellow to red-yellow, covered with pale, thin setae and white scale setae, with pair of longitudinal, dark patches mediolaterally on eye field; fovea oval. Chelicerae yellow, with two promarginal teeth and one retromarginal tooth. Endites colored as chelicerae, with pale inner margins. Labium dark brown, almost linguiform. Sternum almost shield-shaped. Legs pale to yellow except femora I and IV brown to red-brown, with three and two spines on tibiae I and metatarsi I, respectively. Abdomen elongated, dorsum red-brown, with transverse, white stripe of setae medially and several transverse dotted lines posteriorly; venter brown. Palp (Fig. 17A–E): femur longer than wide, with triangular, prolateral apophysis; patella enlarged, longer than wide; tibia wider than long, with disciform VTA in retrolateral view, flat RTA curved anteroventrally, and blunt apically, and sclerotized, triangular DTA; cymbium longer than wide; bulb swollen, with sperm ducts extending along margin; embolus tapered, twisted; MA elongated, with spine-shaped processes on retrolateral portion of distal half and anterior margin.

**Female** (Fig. 18A–C, F). Total length 3.88. Carapace 1.57 long 1.14 wide. Abdomen 2.24 long, 1.10 wide. Clypeus 0.02 high. Eye sizes and inter-distances: AME 0.40, ALE 0.23, PLE 0.22, AERW 1.19, PERW 1.17, EFL 1.02. Legs: I 3.13 (0.95, 0.80, 0.75, 0.38, 0.25), II 2.59 (0.75, 0.40, 0.63, 0.53, 0.28), III 2.69 (0.75, 0.38, 0.63, 0.65, 0.28), IV 3.56 (0.93, 0.50, 0.95, 0.88, 0.30). Habitus (Fig. 18F) similar to that of male except without transverse, white stripe of setae medially on dorsum of abdomen. Epigyne (Fig. 18A–C): slightly wider than long, with broad, medio-posteriorly located, posteriorly opened hood; copulatory openings located anteriorly, separated from each other by less than hood width; copulatory ducts thick, short, with terminal accessory glands, connected to the middle of anterior margins of spermathecae; spermathecae oval, touching, extending anterolaterally; fertilization ducts originate from antero-inner margins of spermathecae, extending transversely.

#### Distribution.

Known only from the type locality in Ninh Binh Province, Vietnam.

### ﻿ Synagelides pengi sp. nov.

Taxon classificationAnimaliaAraneaeSalticidae

2C87AB9B-0EC6-5EF7-8D9F-8CC254184E49

https://zoobank.org/6AD3717E-0CA2-46C3-AD99-21902980E776

Fig. 19

#### Type material.

***Holotype*** ♀ (IZCAS-Ar44265), Vietnam: Ha Giang Province: Vi Xuyen County, Ha Giang National Forest, 14.VIII.2002, D.S. Pham leg. ***Paratypes*** 2♀ (IZCAS-Ar44266–44267), same data as holotype.

#### Etymology.

The species name is a patronym in honor of Prof. Xianjin Peng (Changsha, China), who has made significant contributions to the taxonomy of Chinese salticid spiders; noun (name) in genitive case.

#### Diagnosis.

**Figure 19.**
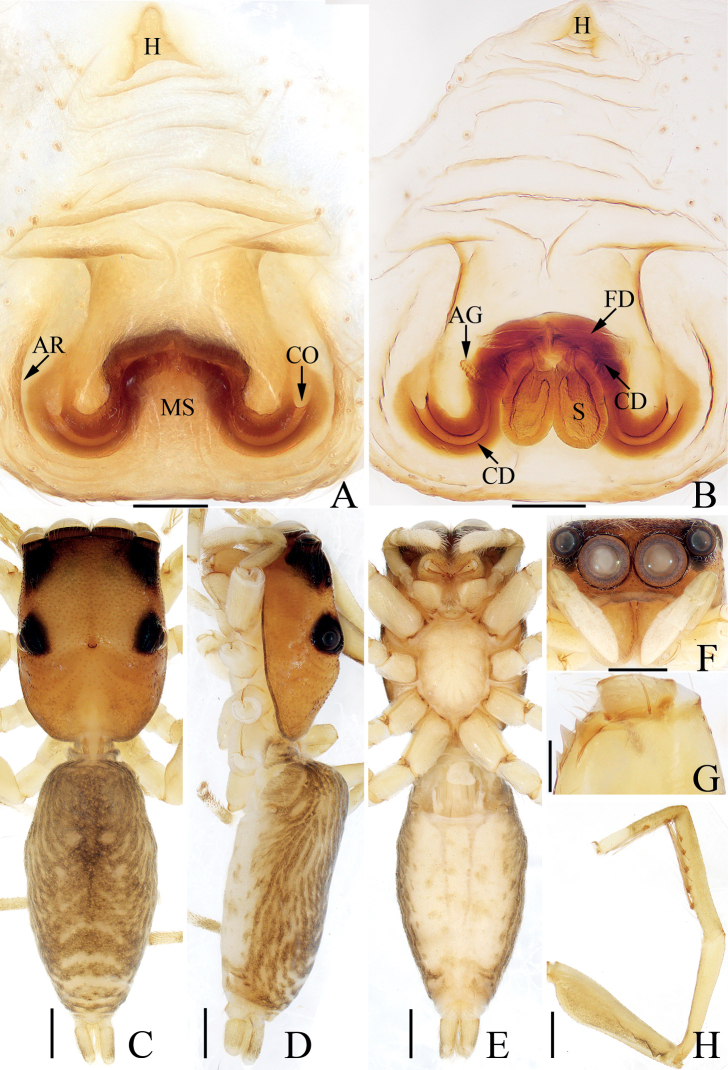
*Synagelidespengi* sp. nov., female holotype **A** epigyne, ventral **B** vulva, dorsal **C** habitus, dorsal **D** ditto, lateral **E** ditto, ventral **F** carapace, frontal **G** chelicera, posterior **H** leg I, retrolateral. Scale bars: 0.1 mm (**A, B, G**); 0.5 mm (**C–F, H**). Abbreviations: AG – accessory gland; AR – atrial ridge; CD – copulatory duct; CO – copulatory opening; FD – fertilization duct; MS – median septum; H – epigynal hood; S – spermatheca.

*Synagelidespengi* sp. nov. resembles *S.yinae* Liu, Chen, Xu & Peng, 2017 in having similarity-shaped copulatory ducts and spermathecae, but it can be easily distinguished by the following: (1) the distance between hood and atrium is almost equal to the atrial length (Fig. 19A), whereas almost equal to one-third the atrial length in *S.yinae* (Liu et al. 2017: figs 7C, 8A); (2) the epigynal hood is almost triangular (Fig. 19A), whereas almost tubiform in *S.yinae* (Liu et al. 2017: figs 7C, 8A).

#### Description.

**Female** (Fig. 19). Total length 5.07. Carapace 2.18 long, 1.59 wide. Abdomen 2.82 long, 1.44 wide. Clypeus 0.04 high. Eye sizes and inter-distances: AME 0.47, ALE 0.29, PLE 0.28, AERW 1.46, PERW 1.62, EFL 1.18. Legs: I 5.61 (1.70, 1.50, 1.43, 0.63, 0.35), II 3.82 (1.13, 0.58, 0.93, 0.80, 0.38), III 4.00 (1.13, 0.53, 0.95, 1.01, 0.38), IV 5.45 (1.50, 0.70, 1.50, 1.35, 0.40). Carapace stippled, yellow, with pair of indistinct, pale brown patches located at anterior 1/3 of eye field, covered with pale thin setae anteriorly; fovea oval, between the PLEs. Chelicerae pale yellow, with two promarginal teeth and one retromarginal tooth. Labium colored as chelicerae, with paler inner margins bearing brown, thin setae. Endites wider than long, covered with several brown setae anteriorly. Sternum nearly shield-shaped. Legs yellow, more or less intermingled with brown, with five and two pairs of ventral spines on tibiae and metatarsi I, respectively. Abdomen elongated, dorsum yellow to brown, with pair of indistinct muscle depressions followed by pair of spots, pair of oblique lines, and several transverse, arc-shaped, pale stripes; venter pale, with pair of brown lines medially on anterior half. Epigyne (Fig. 19A, B): longer than wide, with triangular anterior hood distant from copulatory opening; atrium oval, with pair of arc-shaped posterolateral atrial ridges, separated by a sub-square median septum; copulatory openings located posterolaterally, separated by ~ 3× width of hood; copulatory ducts long, S-shaped, with pair of elongated, terminal accessory glands; spermathecae elongate-oval, ~ 1.5× longer than wide, touching; fertilization ducts originate from anterior portions of spermathecae, curved 90° before extending transversely.

**Male.** Unknown.

#### Distribution.

Known only from the type locality in Ha Giang Province, Vietnam.

#### Comments.

According to morphological features, the species shares a similar habitus and epigyne with *S.forkiforma* Yang, Zhu & Song, 2007, *S.hamatus* Zhu, Zhang, Zhang & Chen, 2005, *S.jingzhao* Yang, Zhu & Song, 2007, *S.latus* Li, Wang & Peng, 2021, *S.lushanensis* Xie & Yin, 1990, *S.triangulus* Li, Wang & Peng, 2021, *S.wuliangensis* Wang, Mi, Irfan & Peng, 2020, and *S.yinae* Liu, Chen, Xu & Peng, 2017, and they could be assigned into an un-described group, recognized by the female having anterior epigynal hood, S-shaped copulatory ducts, oval or elongate-oval spermathecae, and the male palp with spine-shaped RTA and paliform DTA (or BTA described in Li et al. 2021). Despite the fact that the species is only known from females, we are confident in describing it as new because there is no congener known only from males among those species.

### ﻿ Synagelides sancha sp. nov.

Taxon classificationAnimaliaAraneaeSalticidae

0F696DC2-398C-5328-A8A3-854D9B8BBF0E

https://zoobank.org/9394A3E8-661A-41C6-BBB0-FBC09721922F

Figs 20 , 21

#### Type material.

***Holotype*** ♂ (IZCAS-Ar44268), Vietnam: Vinh Phuc Province: Tam Dao National Park (21°31.50'N, 105°33.43'E, ca. 1060 m), 16.V.2007, D.S. Pham leg. ***Paratypes*** 7♀ (IZCAS-Ar44269–44275), same data as holotype; 1♂2♀ (IZCAS-Ar44276–44278), Tam Dao National Park (21°28.34'N, 105°38.09'E, ca. 1010 m), 19.IX.2007, same collector; 2♂2♀ (IZCAS-Ar44279–44282), Hai Phong Province: Cat Ba National Park (20°47.71'N, 107°01.23'E, ca. 130 m), 24.V.2007, same collector; 2♂2♀ (IZCAS-Ar44283–44286), Cat Ba National Park (20°48.26'N, 107°00.58'E, ca. 130 m), 27.VII.2007, same collector; 1♂2♀ (IZCAS-Ar44287–44289), Cat Ba National Park (20°48.08'N, 107°00.21'E, ca. 120 m), 27.VIII.2007, same collector; 3♀ (IZCAS-Ar44290–44292), Cat Ba National Park (20°46.59'N, 107°01.03'E, ca. 100 m), 26.IX.2007, same collector; 1♂ (IZCAS-Ar44293), Cat Ba National Park (20°48.08'N, 107°00.21'E, ca. 120 m), 23.I.2008, same collector.

#### Etymology.

The specific name comes from the Chinese pinyin *san cha* (trident) and refers to the trifurcate RTA; noun.

#### Diagnosis.

**Figure 20.**
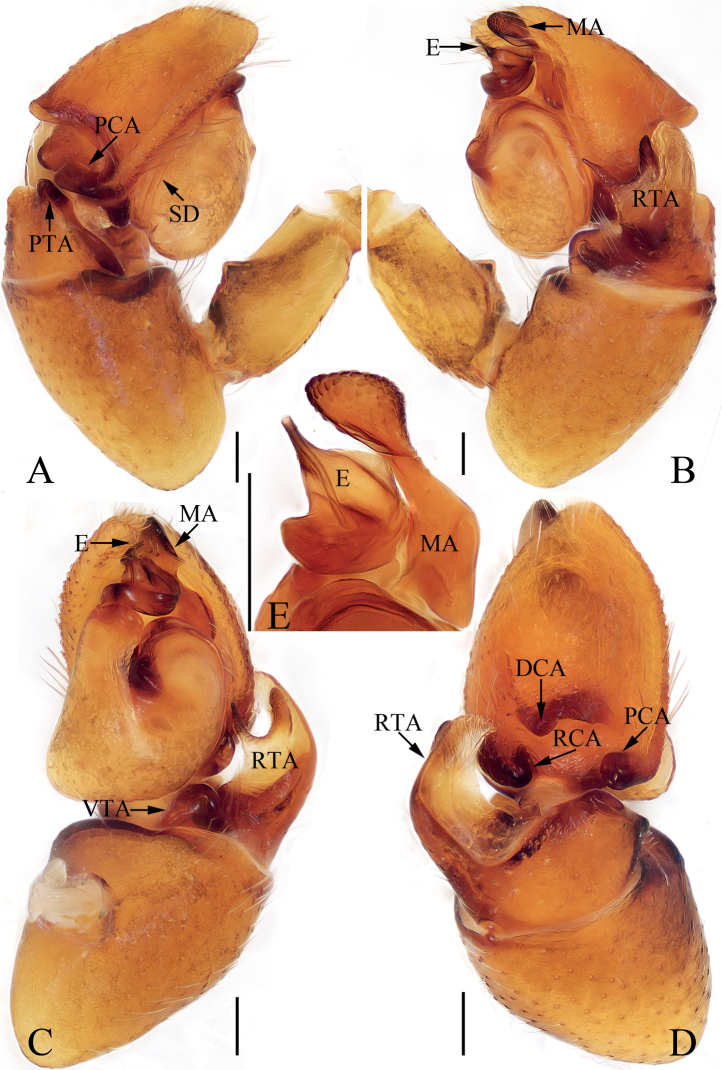
Male palp of *Synagelidessancha* sp. nov., holotype and paratype **A** holotype, prolateral **B** ditto, retrolateral **C** ditto, ventral **D** ditto, dorsal **E** paratype embolus and median apophysis, retrolateral. Scale bars: 0.1 mm. Abbreviations: DCA – dorsal cymbial apophysis; E – embolus; MA – median apophysis; PCA – prolateral cymbial apophysis; PTA – prolateral tibial apophysis; RCA – retrolateral cymbial apophysis; RTA – retrolateral tibial apophysis; SD – sperm duct; VTA – ventral tibial apophysis.

**Figure 21.**
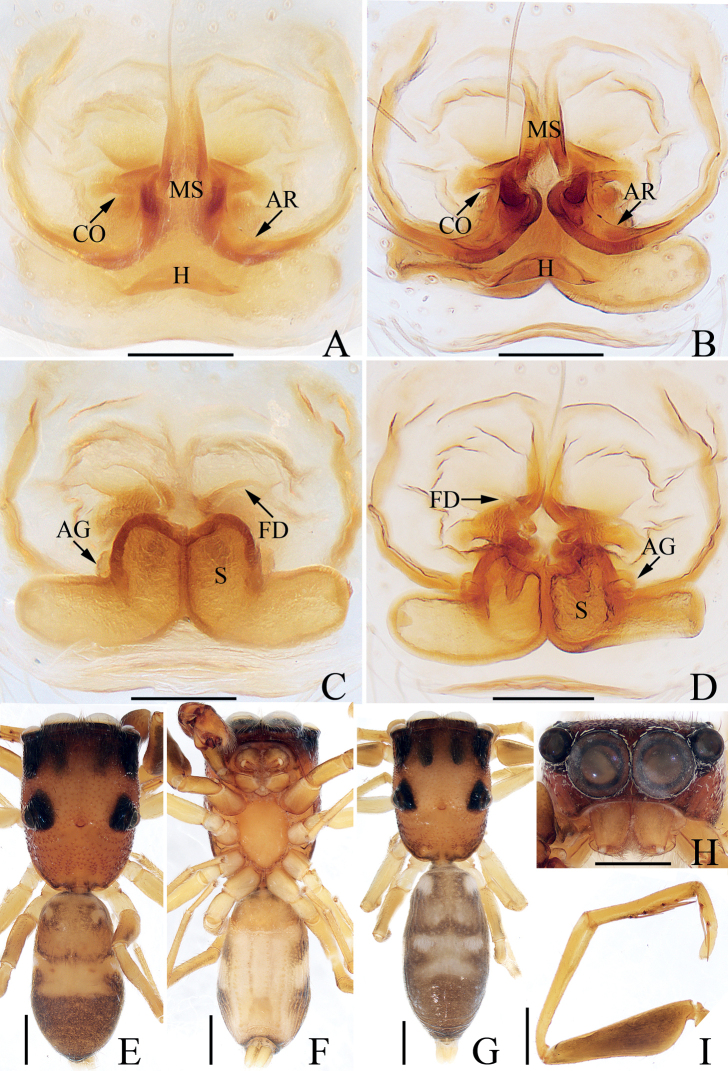
*Synagelidessancha* sp. nov., male holotype and female paratype **A, B** epigyne, ventral **C, D** vulva, dorsal **E** holotype habitus, dorsal **F** ditto, ventral **G** female paratype habitus, dorsal **H** holotype carapace, frontal **I** holotype leg I, prolateral. Scale bars: 0.1 mm (**A–D**); 0.5 mm (**E–I**). Abbreviations: AG – accessory gland; AR – atrial ridge; CO – copulatory opening; FD – fertilization duct; MS – median septum; H – epigynal hood; S – spermatheca.

The male of *Synagelidessancha* sp. nov. can be easily distinguished from other congeners by the trifurcate RTA (Fig. 20B, C), whereas it is bifurcated or lacking branches in other species (Metzner 2023). The female of this new species resembles *S.subgambosus* Wang, Mi, Irfan & Peng, 2020 in having a similar epigyne, but it can be distinguished by the following: (1) the presence of a posterior epigynal hood (Fig. 21A, B), which is lacking and instead replaced by an epigynal fold in the same position in *S.subgambosus* (Wang et al. 2020: fig. 12A); (2) the visible parts of the accessory glands are shorter than 1/8 of the spermathecal width (Fig. 21C, D), whereas they are ~ 2/3 of the spermathecal width in *S.subgambosus* (Wang et al. 2020: fig. 12C).

#### Description.

**Male** (Figs 20, 21E, F, H, I). Total length 3.35. Carapace 1.63 long, 1.20 wide. Abdomen 1.71 long, 0.96 wide. Clypeus 0.03 high. Eye sizes and inter-distances: AME 0.44, ALE 0.25, PLE 0.20, AERW 1.20, PERW 1.19, EFL 0.96. Legs: I 4.43 (1.38, 1.33, 1.01, 0.43, 0.28), II 2.79 (0.83, 0.43, 0.65, 0.58, 0.30), III 2.84 (0.83, 0.40, 0.63, 0.68, 0.30), IV 3.68 (1.05, 0.50, 0.95, 0.88, 0.30). Carapace stippled, red-yellow to dark, with pair of longitudinal, mediolateral, dark stripes on anterior half of eye field, covered with white and brown setae; fovea oval, dark red. Chelicerae yellow, with two promarginal teeth and one retromarginal tooth. Endites almost square, bearing dense setae on pale margins. Labium pale. Sternum almost shield-shaped. Legs yellow except femora I red-brown, with seven (2-2-2-1) and two ventral spines on tibiae and metatarsi I, respectively. Abdomen elongated, dorsum yellow to brown, with pair of pale yellow spots anteriorly, and large, irregular transverse patch medially; venter pale, with longitudinal dotted lines. Palp (Fig. 20A–E): femur longer than wide, with triangular disto-prolateral apophysis; patella enlarged, ~ 1.4× longer than wide in retrolateral view; tibia short, with sub-triangular prolateral apophysis, disciform ventral apophysis, trifurcated retrolateral apophysis; cymbium longer than wide, with flat, baso-prolateral apophysis, irregularly-shaped dorsal apophysis, and short, blunt retrolateral apophysis; bulb swollen, with sperm duct extending along margin; embolus originates from antero-prolateral side of bulb, curved into a circle, acutely narrowed distally; MA elongated, retrolateral to embolus, bearing small tubercles distally.

**Female** (Fig. 21A–D, G). Total length 3.71. Carapace 1.57 long, 1.17 wide. Abdomen 2.07 long, 1.02 wide. Clypeus 0.03 high. Eye sizes and inter-distances: AME 0.45, ALE 0.25, PLE 0.21, AERW 1.21, PERW 1.20, EFL 0.97. Legs: I 3.44 (1.05, 1.01, 0.75, 0.38, 0.25), II 2.54 (0.75, 0.40, 0.58, 0.53, 0.28), III 2.59 (0.70, 0.38, 0.58, 0.65, 0.28), IV 3.49 (0.93, 0.45, 0.93, 0.88, 0.30). Carapace similar to that of male except paler. Abdomen gray-brown, with pairs of antero-marginal and mediolateral white spots of setae, and irregularly-shaped, median pale patch. Epigyne (Fig. 21A–D): wider than long, with broad, boat-shaped hood opening posteriorly; atria oval, occupying 2/3 of epigynal surface, separated by tapered median septum, with pair of arc-shaped lateral ridges; copulatory openings small, near the base of median septum, copulatory ducts very short, mostly hidden by spermathecae, with terminal accessory glands; spermathecae L-shaped, touching; fertilization ducts originate from anterior portions of the longitudinal parts of spermathecae, extending anterolaterally.

#### Distribution.

Known only from the type locality in Vinh Phuc and Hai Phong Provinces, Vietnam.

### ﻿Genus Yaginumaella

Taxon classificationAnimaliaAraneaeSalticidae

Prószyński, 1979

D6387533-D568-5464-A76D-510D019F6D0A

#### Type species.

*Pellenesususudi* Yaginuma, 1972 from Japan.

#### Comments.

*Yaginumaella*, contains 13 species mainly restricted to East Asia (WSC 2023). It was represented by nearly 50 species before Patoleta et al. (2020) transferred 37 of them into *Ptocasius*. However, the decision to transfer the species requires further confirmation because it is only based on the similarity of copulatory organs and ignores habitus differences. Additionally, there are no molecular data to provide another line of evidence for this hypothesis. To date, there are no *Yaginumaella* species recorded from Vietnam. Our experience indicates that there are quite a few species that are yet to be discovered from the unexplored, high-altitude mountains of South China, and the true diversity of the genus will greatly increase.

### ﻿ Yaginumaella hagiang sp. nov.

Taxon classificationAnimaliaAraneaeSalticidae

F6EBCFD6-C1A3-5106-89F5-7CE22CBAB405

https://zoobank.org/2F56F12D-D056-4DBC-8934-099F6FA877C6

Figs 22 , 23

#### Type material.

***Holotype*** ♂ (IZCAS-Ar44294), Vietnam: Ha Giang Province: Ha Giang National Forest, 14.VII.2002, D.S. Pham leg. ***Paratype*** 1♂ (IZCAS-Ar44295), same data as holotype.

#### Etymology.

The species is named after the type locality; noun in apposition.

#### Diagnosis.

**Figure 22.**
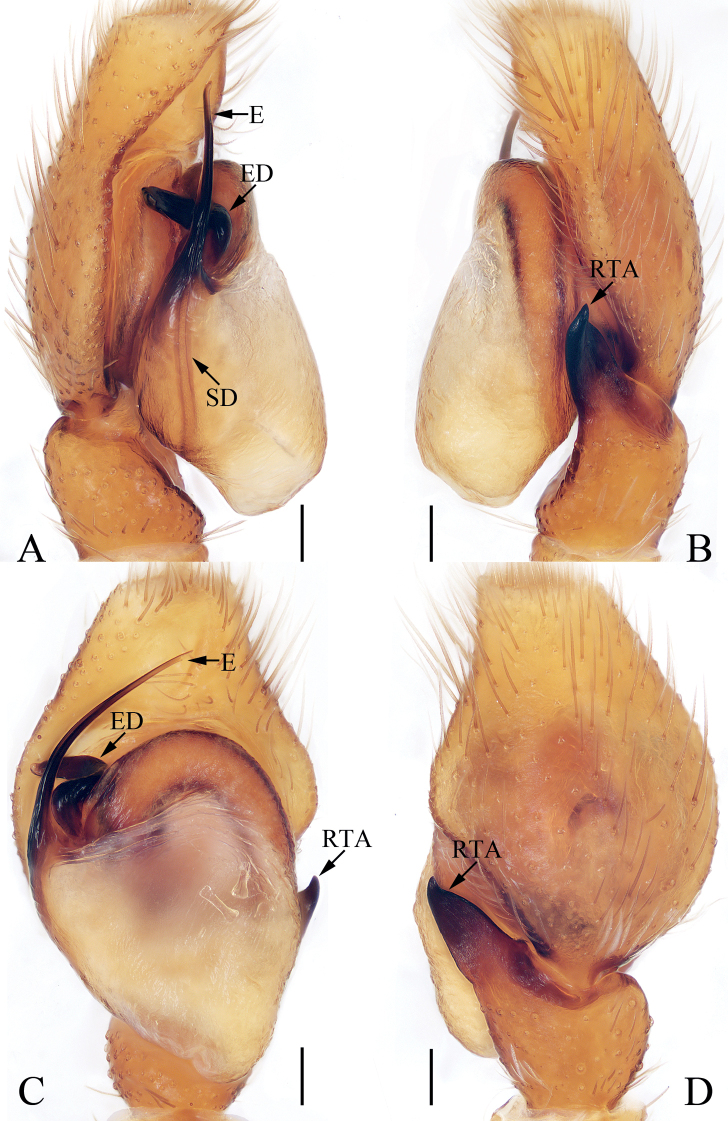
Male palp of *Yaginumaellahagiang* sp. nov., holotype **A** prolateral **B** retrolateral **C** ventral **D** dorsal. Scale bars: 0.1 mm. Abbreviations: E – embolus; ED – embolic division; RTA – retrolateral tibial apophysis; SD – sperm duct.

*Yaginumaellahagiang* sp. nov. closely resembles *Y.orthomargina* Shao, Li & Yang, 2014 in having a very similar palpal structure, but it can be distinguished by the following: (1) the tip of embolic division is directed prolaterally (Fig. 22C), whereas it is directed anteriorly in *Y.orthomargina* (Shao et al. 2014: fig. 10); (2) the RTA is slightly curved towards the dorsal side distally in retrolateral view (Fig. 22B), whereas it is straight in *Y.orthomargina* (Shao et al. 2014: fig. 11).

#### Description.

**Figure 23.**
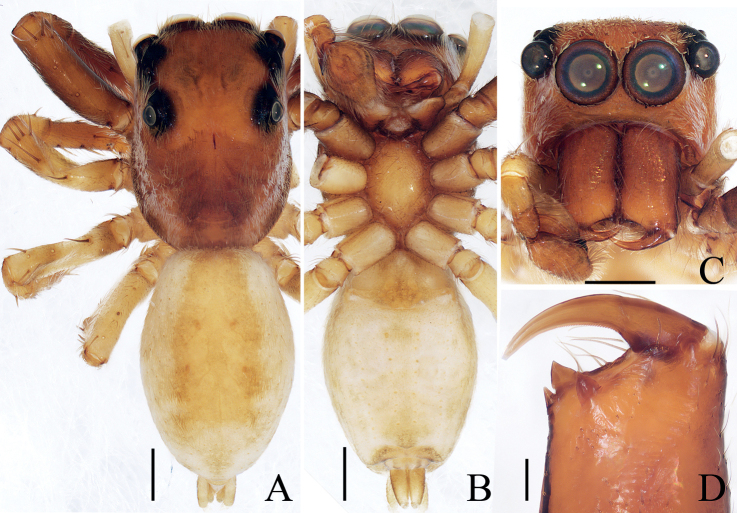
*Yaginumaellahagiang* sp. nov., holotype **A** habitus, dorsal **B** ditto, ventral **C** carapace, frontal **D** chelicera, posterior. Scale bars: 0.1 mm (**D**); 0.5 mm (**A–C**).

**Male** (Figs 22, 23). Total length 4.43. Carapace 2.24 long, 1.59 wide. Abdomen 2.27 long, 1.53 wide. Clypeus 0.10 high. Eye sizes and inter-distances: AME 0.43, ALE 0.27, PLE 0.25, AERW 1.39, PERW 1.39, EFL 0.94. Legs: I 4.41 (1.33, 0.70, 1.03, 0.75, 0.60), II 3.72 (1.05, 0.68, 0.83, 0.63, 0.53), III 4.37 (1.30, 0.63, 0.90, 1.01, 0.53), IV 4.50 (1.30, 0.63, 1.01, 1.01, 0.55). Carapace red-brown, setose, with indistinct patches medially on eye field; fovea dark, longitudinal, linear. Chelicerae red-brown, with two promarginal teeth and one retromarginal tooth. Endites colored as chelicerae, broadened distally. Labium tapered, darker than endites. Sternum yellow to red-brown, covered with brown setae. Legs yellow to red-brown, spinous. Abdomen elongate-oval, dorsum pale to yellow, covered with large scutum extending from anterior margin to posterior 3/4, and ~ 1/2 the abdominal width; venter pale, dotted. Palp: (Fig. 22A–D): tibia slightly longer than wide in retrolateral view, with broad retrolateral apophysis ~ 2/3 of its length, slightly curved distally, with pointed tip; cymbium setose; bulb swollen, with sperm duct extending along margin; embolus originates from 9 o’clock position on bulb, slightly curved medially, with strongly sclerotized, hook-shaped division.

**Female.** Unknown.

#### Distribution.

Only known from the type locality in Ha Giang Province, Vietnam.

#### Comments.

The species is known only from males, but there are no other congeners known only from single females distributed in the nearby area, so, there is a very small possibility that the species belongs to a species that is already described only known from females. Thus, we describe it as new herein.

### ﻿Genus Zabka gen. nov.

Taxon classificationAnimaliaAraneaeSalticidae

99A81B8C-2D44-5540-A40C-E3F8E4DF9E31

https://zoobank.org/59A077F9-1DB3-4009-8477-9DA0CD65CEF2

#### Type species.

*Euophryscooki* Żabka, 1985 from Vietnam.

#### Etymology.

The genus is named after Prof. Marek Żabka (Siedlce, Poland), a leading arachnologist in jumping spiders, who contributed significantly to the taxonomy of jumping spiders from Vietnam. The gender is masculine.

#### Diagnosis.

*Zabka* gen. nov. resembles that of *Euochin* Prószyński, 2018 in having the indistinguishable male palp, and the big, round, paired atria, but it can be distinguished by the following: (1) the male chelicerae have one retromarginal fissidental tooth with one cusp, or a single tooth, whereas have one fissidental tooth with multiple cusps in the generotype and its congeners of *Euochin* (see the description of Zha et al. 2014); (2) the bulb has small, antero-marginal lobe, whereas absent in *Euochin* (Zha et al. 2014: figs 5, 6, 8, 9, 16, 17, 19, 20; Metzner 2023); (3) the spermathecae are anterior located, whereas posteriorly located in *Euochin* (Zha et al. 2014: figs 4, 11, 15, 22; Metzner 2023); (4) the copulatory openings are located medially or posteriorly on atria, whereas anteriorly located, or beneath the anterior portions of atrial ridges in *Euochin* (Zha et al. 2014: figs 3 10, 14, 21; Metzner 2023); (5) the copulatory ducts are much longer, > 3× spermathecal diameter, whereas usually less than the spermathecal diameter in *Euochin* (Zha et al. 2014: figs 4, 11, 15, 22; Metzner 2023). The genus also somewhat resembles *Euophrys* C. L. Koch, 1834 in male palpal structure, but it can be easily distinguished by the broad RTA (in retrolateral view), whereas RTA is seta-like in *Euophrys* (see the description of Prószyński et al. 2018).

#### Description.

Small-sized spiders. Sexual dimorphism indistinct. Carapace almost square, red-brown to dark brown, setose, with elevated cephalic region and sloped thorax; fovea dark, longitudinal, bar-shaped. Chelicerae with two promarginal teeth and one retromarginal fissidental tooth or single tooth. Endites broadened distally. Labium almost linguiform. Sternum almost oval, with straight anterior margin. Legs yellow to dark brown. Abdomen oval or elongate-oval, dorsum with alternate pale yellow and dark brown transverse bands or with several chevrons posteriorly; venter yellow-brown to dark brown. Palp: tibia short, with ventral bump, and straight retrolateral apophysis almost equal to its length; cymbium longer than wide; bulb elongated, with posteriorly extending posterior lobe and small, antero-marginal lobe; embolus originates from the anterior portion of bulb, forming a disc at base, coiled in almost a circle. Epigyne: with big, round, paired atria; copulatory openings medially or posteriorly located on atria; copulatory ducts long, coils almost C-shaped, with proximal accessory glands or not; spermathecae almost round, spherical, anteriorly located; fertilization ducts originate from the anterior margins of spermathecae, transversely extending.

#### Composition.

The genus belongs to the tribe Euophryini, only including the generotype and *Z.xuyei* (Lin & Li, 2020), comb. nov.

#### Distribution.

Vietnam (Nghe An, Ninh Binh), China (Yunnan).

#### Comments.

According to the morphological features, it can be easily recognized that *Euophrysxuyei* is not a true *Euophrys*. Herein it is being transferred into *Zabka* gen. nov. based on the similarity of copulatory organs with the generotype. However, it is also different from the latter in habitus markings, indicating that the generic position of this species may need further confirmation.

### ﻿ Zabka cooki

Taxon classificationAnimaliaAraneaeSalticidae

(Żabka, 1985) comb. nov.

55800303-C11E-5B7E-8926-8F013249FF79

Figs 24 , 25

Euophrys cooki Żabka, 1985: 219, figs 149, 150; Logunov 2020: 560.
“Euophrys” cooki: Prószyński et al. 2018: 38, fig. 17I.

#### Material examined.

1♀ (IZCAS-Ar44296), Vietnam: Ninh Binh Province: Cuc Phuong National Park, Disturbed Forest (20°17.07'N, 105°40.25'E, ca. 270 m), 3.IV.2007, D.S. Pham leg.; 1♀ (IZCAS-Ar44297), Cuc Phuong National Park, 1–30.VI.2007, same collector; 1♂6♀ (IZCAS-Ar44298–44304), Cuc Phuong National Park, 1–30.VII.2007, same collector; 5♀ (IZCAS-Ar44305–44309), Cuc Phuong National Park, 1–30.X.2007, same collector; 1♀ (IZCAS-Ar44310), Cuc Phuong National Park (20°21.44'N, 105°34.21'E, ca. 410 m), 8.X.2007, same collector; 1♀ (IZCAS-Ar44311), Cuc Phuong National Park (20°15.30'N, 105°42.55'E, ca. 250 m), 9.X.2007, same collector; 3♀ (IZCAS-Ar44312–44314), Cuc Phuong National Park, 1–30.I.2008, same collector.

#### Diagnosis.

**Figure 24.**
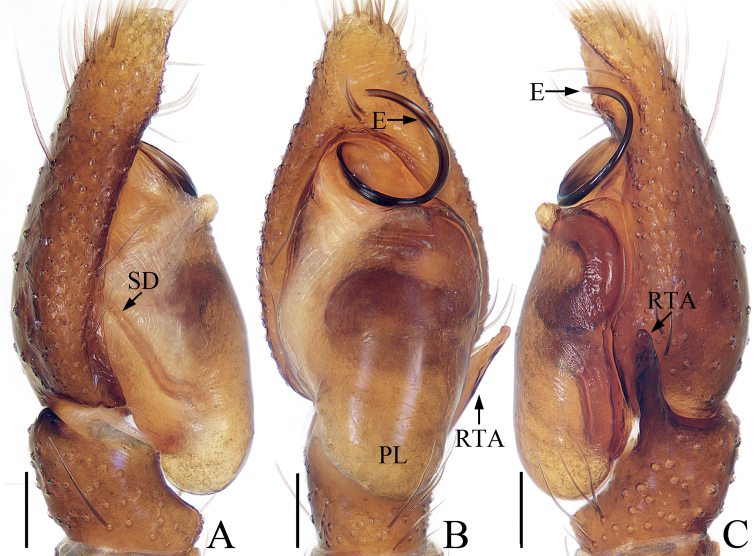
Male palp of *Zabkacooki*, comb. nov. **A** prolateral **B** ventral **C** retrolateral. Scale bars: 0.1 mm. Abbreviations: E – embolus; PL – posterior lobe; RTA – retrolateral tibial apophysis; SD – sperm duct.

**Figure 25.**
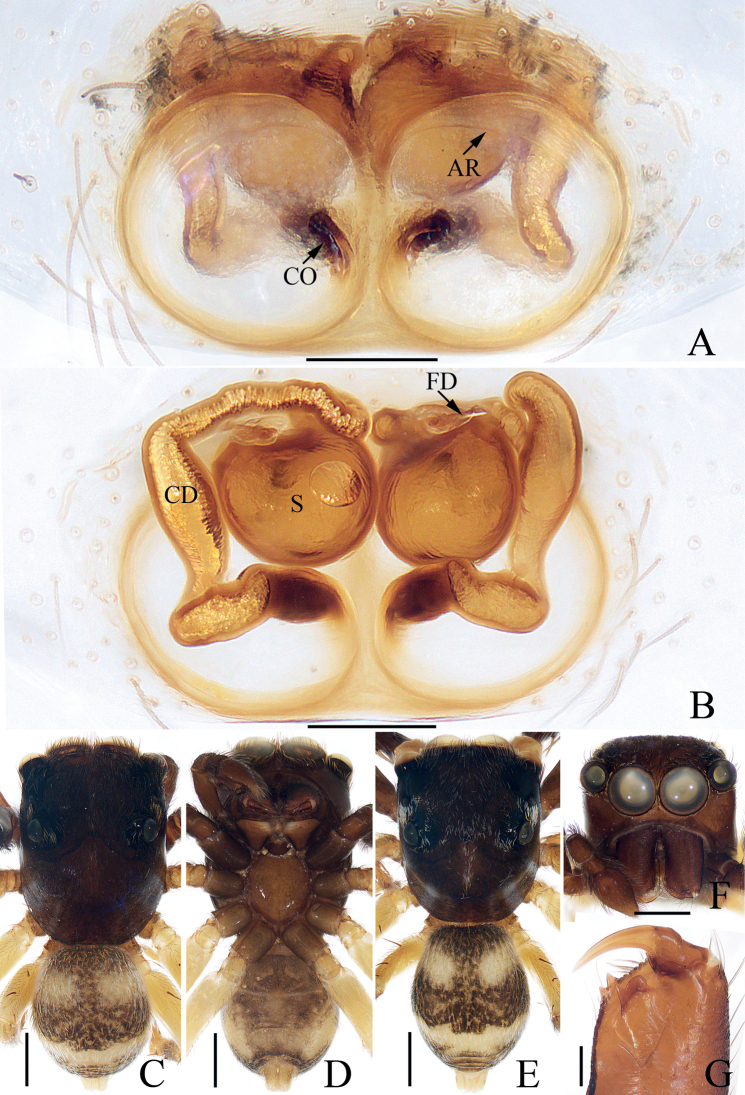
*Zabkacooki*, comb. nov. **A** epigyne, ventral **B** vulva, dorsal **C** male habitus, dorsal **D** ditto, ventral **E** female habitus, dorsal **F** male carapace, frontal **G** male chelicera, posterior. Scale bars: 0.1 mm (**A, B, G**); 0.5 mm (**C–F**). Abbreviations: AR– atrial ridge; CD – copulatory duct; CO – copulatory opening; FD – fertilization duct; S – spermatheca.

*Zabkacooki* resembles *Z.xuyei*, comb. nov. in having similar copulatory organs, but it can be easily distinguished by the following: (1) the mediodorsal cymbial spines are absent (Fig. 24A, C), whereas the spines are present in *Z.xuyei* (Lin and Li 2020: fig. 5A, C); (2) the copulatory ducts are twisted proximally and lack accessory glands (Fig. 25B), whereas they are not twisted and have proximal accessory glands in *Z.xuyei* (Lin and Li 2020: fig. 6B); (3) the dorsum of abdomen has a pair of anterior, elongate-oval, pale yellow patches and a transverse, posterior, pale yellow band in both sexes (Fig. 25C, E), whereas there are several chevrons in *Z.xuyei* (Lin and Li 2020: fig. 6C, E).

#### Description.

**Male** (Figs 24, 25C, D, F, G). Total length 3.01. Carapace 1.82 long, 1.40 wide. Abdomen 1.24 long, 1.10 wide. Clypeus 0.08 high. Eye sizes and inter-distances: AME 0.44, ALE 0.29, PLE 0.25, AERW 1.41, PERW 1.23, EFL 0.85. Legs: I 3.99 (1.25, 0.63, 1.10, 0.63, 0.38), II 2.83 (0.90, 0.50, 0.68, 0.45, 0.30), III 3.61 (1.25, 0.53, 0.75, 0.78, 0.30), IV 3.51 (1.13, 0.50, 0.75, 0.80, 0.33). Carapace red to dark brown, covered with dense, brown setae, with square cephalic region bearing two pairs of scale setae, one pair between PLEs and PMEs and other pair near the posterior-inner margins of PMEs; fovea dark, longitudinal, linear. Chelicerae red-brown to dark brown, with two promarginal teeth and one retromarginal fissidental tooth with one cusp. Endites paler than chelicerae, broadened distally. Labium tapered, paler at distal end. Sternum almost oval, with sub-straight anterior margin. Legs pale yellow to yellow except femora I dark brown, with three and two pairs of ventral spines on patellae I and metatarsi I, respectively. Abdomen oval, dorsum pale yellow to dark brown, covered with white setae, with pair of anterolateral, elongate-oval, pale yellow patches and transverse, posterior pale yellow band; venter pale yellow to brown. Palp (Fig. 24A–C): tibia longer than wide in retrolateral view, with medio-posterior notch, and straight RTA almost equal to its length and blunt apically; cymbium longer than wide, and tapered at distal half; bulb elongated, with sperm duct strongly curved at anterior half, with oval anterior lobe, and well-developed, posteriorly extended posterior lobe; embolus originates from the antero-prolateral portion of bulb, forming disc at base, remainder curved into nearly a complete circle, with blunt tip.

**Female** (Fig. 25A, B, E). Total length 2.99. Carapace 1.59 long, 1.32 wide. Abdomen 1.37 long, 1.10 wide. Clypeus 0.08 high. Eye sizes and inter-distances: AME 0.44, ALE 0.29, PLE 0.24, AERW 1.37, PERW 1.21, EFL 0.89. Legs: I 3.24 (1.01, 0.55, 0.80, 0.55, 0.33), II 2.61 (0.80, 0.50, 0.58, 0.45, 0.28), III 3.33 (1.20, 0.50, 0.70, 0.65, 0.28), IV 3.26 (1.10, 0.43, 0.70, 0.73, 0.30). Habitus (Fig. 25E) similar to that of male except darker and with denser white setae on carapace. Epigyne (Fig. 25A, B) wider than long, with pair of round atria separated from each other by < 1/6 of their width; copulatory openings slit-shaped, located at middle of inner portions of atria, separated from each other by ~ 1/4 of the atrial width; copulatory ducts long, extending transversely in opposite directions before ascending to the same level of anterior margins of spermathecae, finally connecting to anterior portion of spermathecae; spermathecae almost spherical, anteriorly located, touching each other; fertilization ducts originate from the middle of anterior margins of spermathecae, extending transversely.

#### Distribution.

Vietnam (Nghe An, Ninh Binh).

## Supplementary Material

XML Treatment for Chinattus

XML Treatment for Chinattus crewsae

XML Treatment for Chinattus logunovi

XML Treatment for Eupoa

XML Treatment for Eupoa maidinhyeni

XML Treatment for Eupoa maddisoni

XML Treatment for Eupoa ninhbinh

XML Treatment for Eupoa zabkai

XML Treatment for Indopadilla

XML Treatment for Indopadilla cuc

XML Treatment for Synagelides

XML Treatment for Synagelides ani

XML Treatment for Synagelides mii

XML Treatment for Synagelides pengi

XML Treatment for Synagelides sancha

XML Treatment for Yaginumaella

XML Treatment for Yaginumaella hagiang

XML Treatment for Zabka

XML Treatment for Zabka cooki
